# Genetic and Phenotypic Diversity and Evaluation of Total Phenolics and Antioxidant Properties of Garlic Landraces from Lazio Region (Central Italy): “Aglio Rosso di Proceno” and “Aglio Rosso di Castelliri”

**DOI:** 10.3390/plants14081189

**Published:** 2025-04-11

**Authors:** Enrica Alicandri, Diana De Santis, Margherita Modesti, Serena Ferri, Anna Rita Paolacci, Roberto Rea, Mario Ciaffi

**Affiliations:** 1 Department for Innovation in Biological, Agro-Food and Forest Systems (DIBAF), University of Tuscia, Via San Camillo De Lellis snc, 01100 Viterbo, Italy; enrica.alicandri@unitus.it (E.A.); desdiana@unitus.it (D.D.S.); margherita.modesti@unitus.it (M.M.); serenaferri@unitus.it (S.F.); arpaolacci@unitus.it (A.R.P.); 2 ARSIAL, Regional Agency for the Development and the Innovation of Lazio Agriculture, Via Rodolfo Lanciani 38, 00162 Rome, Italy; r.rea@arsial.it

**Keywords:** garlic landraces, SSR and ISSR markers, genetic diversity, population structure, phenotypic traits, phenolic content

## Abstract

Garlic (*Allium sativum* L.) is the second most significant species within the *Allium* genus worldwide, widely used in cooking and both traditional and modern medicine due to its beneficial biological and therapeutic properties. In Italy, diverse pedo-climatic conditions and historical–cultural fragmentation have led to the development of various garlic landraces, prized for their unique organoleptic qualities and cultural importance. This study aimed to assess the intra-varietal diversity and uniqueness of two red garlic landraces from the Lazio region in central Italy, “Aglio Rosso di Castelliri” and “Aglio Rosso di Proceno”, using SSR and ISSR molecular markers, along with evaluations of bulb morphological traits, total phenolic content, and antioxidant properties. The molecular analysis included 11 accessions of “Aglio Rosso di Castelliri”, nine of “Aglio Rosso di Proceno”, and 15 control accessions, comprising eight Italian red-type garlic landraces, four Spanish red garlic commercial varieties, two white garlic accessions, and an accession of *A. ampeloprasum* var. *holmense* used as an outgroup. SSR and ISSR markers revealed moderate genetic diversity within the collection, with mean PIC values of 0.41 and 0.17, respectively. The molecular data identified four distinct genetic clusters, with the two Lazio landraces forming separate groups, indicating their genetic distinctiveness. The results from the STRUCTURE analysis support the hypothesis that these landraces may have originated from the widely cultivated “Aglio Rosso di Sulmona” or a common ancestral population once prevalent in central Italy. The study also revealed significant intra-population genetic diversity within the two garlic landraces, underscoring the need for in situ conservation and clonal selection. Phenotypic evaluations confirmed the distinctiveness of the two landraces, with “Aglio Rosso di Castelliri” characterized by smaller bulbs and cloves with higher dry matter content and distinct color profiles. Additionally, significant variation in total phenolic content and antioxidant activity was observed by analyzing 13 selected accessions from the two landraces (six from “Aglio Rosso di Proceno” and seven from “Aglio Rosso di Castelliri”) and five red garlic control accessions, with the two Lazio landraces exhibiting higher levels than the control group. This study highlights the importance of integrating molecular, phenotypic, and chemical analyses to understand garlic landrace diversity, with significant implications for their conservation and protection of local agro-food products.

## 1. Introduction

Garlic (*Allium sativum* L.) is a diploid bulb species (2n = 2x = 16) belonging to the family Amaryllidaceae in the order Asparagales, which is thought to have originated 10,000 years ago in Central Asia (Kazakhstan, Uzbekistan, Kyrgyzstan, Turkmenistan, Tajikistan, Western China) from its wild ancestor, *Allium longicuspis* [1,2]. It spread early to China and India in the east, and to the Mediterranean basin in the west [2]. Indeed, its secondary center of origin is the Mediterranean and Caucasus regions [3].

Garlic is one of the oldest known crops, with records of its use dating back to 5000 years ago in India and ancient Egypt [4]. It is widely employed in cuisine for flavoring and seasoning, as well as in traditional medicine across the globe. Numerous studies have examined the health benefits of garlic consumption, including reduced risk factors for cancer and cardiovascular disease, immune stimulation, antimicrobial effects [5,6,7,8], stress resistance, and potential anti-aging effects [9,10]. These physiological properties depend on both the typical profile of the bioactive components of garlic and their combined action [11]. The primary bioactive compounds encompass saponins, phenolics, organic acids, and diverse organosulfur compounds [12]. The latter are derived from alliin, which is metabolized into allicin by the enzyme alliinase. Allicin and other sulfoxides undergo numerous transformations, resulting in a broad spectrum of organosulfur volatiles, which are well known not only for their beneficial health effects but also for their distinctive odor and flavor [13,14,15]. Although variations in the bioavailability and bioactivity of saponins and phenolics have been documented, these compounds may also contribute to the antioxidant and anti-inflammatory properties of garlic [12]. Notably, the overall phenolic content in garlic bulbs exhibits a significant positive correlation with antioxidant activity, regardless of variations in the composition of specific phenolic compounds [16,17].

Garlic is cultivated in temperate climates worldwide, with an annual production in 2022 of approximately 28 million tons on about 1.6 million hectares [18]. China and India are the leading producers of garlic in the world (20 and 3 million tons, respectively), accounting for more than 80% of the global production [18]. Garlic output in the European Union accounts for only 3% of total global production [18]. Italian production is approximately 28,000 tons, with the majority of production concentrated in the Campania, Sicily, Veneto, and Emilia-Romagna regions [19]. Garlic imports into Italy have gradually increased in recent years, reaching approximately 30,000 tons in 2022 [18]. In addition to market requirements related to the seasonality of production, the increase in imports is also the result of the inadequate promotion and commercial valorization of Italian products, including those based on landraces.

Garlic, being a sterile species, is predominantly propagated asexually through its bulblets (cloves) due to obligatory apomixis [3]. However, the vegetative method of production has not hampered the ability of the species to accumulate variability, and wide ranges in phenotypic diversity and environmental-adaptation capability have been observed [4,20,21]. The variation observed today in domesticated garlic primarily arises from somatic mutations occurring over the course of extensive cultivation [4], as well as from variability inherited from its wild progenitor [3]. This diversity is spread along geographical and environmental gradients, which are associated with the conservation and distinctiveness of garlic ecotypes [22]. Some of these materials have been maintained in restricted areas and have become valuable landraces (also known as ‘local varieties’ and ‘farmer’s varieties’).

Landraces are distinct but variable populations, usually recognized by a local name, and highly adapted to the environmental conditions of the area where they grow [23]. They are predominantly cultivated in marginal areas with low-input production systems and possess advantageous adaptation traits to a variety of stressful environments. In addition, they are intricately intertwined with the traditions and cultures of the people who have developed and cultivated them for many years [23]. Although landraces represent a significant element of agrobiodiversity, the majority of them are currently at risk of genetic erosion because they are predominantly cultivated by older farmers and are being gradually replaced by modern varieties [23]. Increasing market interest in traditional food products derived from more sustainable production systems has led to a strong re-evaluation and recovery of many traditional crops and neglected genotypes, including a number of garlic landraces [24,25].

In Italy, as in the rest of southern Europe, many garlic landraces are still cultivated for family consumption or niche markets. These landraces have been maintained for their organoleptic attributes, as well as for cultural or sentimental reasons [26,27,28]. The cultivated landraces of garlic in Italy are primarily of two types: white garlic and red garlic [26]. They are characterized by white-silvered and pinkish-purple tunics, respectively. Most of them have been awarded quality marks as protected denomination of origin (PDO) (e.g., “Aglio Polesano” and “Aglio di Voghiera”), Protected Geographical Indication (PGI) (e.g., “Aglio Piacentino”), or belong to the Slow Food Presidium (e.g., “Aglio Rosso di Nubia”, “Aglio di Vessalico”, and “Aglio di Caraglio”). Moreover, some local landraces, such as “Aglio Piacentino” and “Aglio Rosso di Sulmona”, which were originally produced in a restricted geographical area, have spread throughout the country due to their organoleptic properties and long tradition, and have been registered in the “Common catalogue of varieties of vegetable species” [26,27].

Two landraces of red garlic, “Aglio Rosso di Proceno” and “Aglio Rosso di Castelliri”, are still cultivated in the Lazio region of central Italy. Their production is included among the “Traditional Agri-food Products” (TAPs), and the two landraces are listed in the Regional Voluntary Register (https://www.arsial.it/biodiversita/registro-volontario-regionale/; accessed on 1 September 2024) in accordance with Regional Law No. 15 (1 March 2000), “Protection of genetic resources of agricultural interest, indigenous in Lazio and at risk of erosion” [29]. This regional law entrusts ARSIAL (Regional Agency for Agricultural Development and Innovation of Lazio) with the management of the Voluntary Regional Register. These landraces are named after the localities where they are cultivated: Proceno and Castelliri, two small villages in the provinces of Viterbo (in the north-west of the Lazio region) and Frosinone (in the southeast of the region), respectively. Nowadays, various farmers in the two municipalities grow these landraces in small plots using low-input agronomic practices, such as minimum soil tillage, manual weed control, and minimal or no irrigation, fertilizers, and pesticides. The majority of production is for family use or sold directly to consumers or restaurants. Farmers maintain their own “seed” and have minimal exchange between them. They describe a certain amount of phenotypic variability within each landrace, but without a significant alteration of its characteristic sensory profile.

Garlic germplasm accessions, particularly local landraces, exhibit substantial variability in morphological, agronomic, chemical, and nutritional traits [22,28,30,31,32,33,34]. This variability is crucial for breeding programs; therefore, genetic research on these accessions and landraces is essential for understanding the nature of the variation and ensuring their effective use in developing new varieties [35,36].

Early studies on garlic germplasm diversity primarily focused on morphological traits [22,33,37,38,39]. However, because these traits are influenced by environmental factors, they may not accurately reflect the underlying genetic diversity [35,40]. In contrast, molecular markers are recognized as powerful tools for identifying, characterizing, and evaluating genetic diversity, as they are minimally affected by factors such as age and physiological condition of samples, and environmental conditions [39,40]. As a result, a wide range of molecular markers, including Random Amplified Polymorphic DNA (RAPD) [26,41,42], Sequence-Related Amplified Polymorphisms (SRAPs) [43], Amplified Fragment Length Polymorphisms (AFLPs) [44,45,46], Insertions–Deletions (InDels) [47], Inter-Simple Sequence Repeats (ISSRs) [35,36,48,49], and Simple Sequence Repeats (SSRs) [30,35,50,51,52,53,54,55,56,57], have been progressively used for garlic germplasm identification, crop improvement, and genetic diversity assessment. Among these, SSRs are particularly favored due to their co-dominance, reproducibility, high polymorphism, and cross-species transferability [35,40,55]. ISSRs, on the other hand, are widely used in genetic studies for their informativeness, reliability, and efficiency in laboratory applications [36,48].

This study aimed to evaluate the intra-varietal diversity and distinctiveness of the two previously mentioned red garlic landraces, “Aglio Rosso di Castelliri” and “Aglio Rosso di Proceno”, using SSR and ISSR molecular markers. The evaluation also included assessments of bulb morphological traits, total phenolic content, and antioxidant properties. Although several studies have investigated the morphological, organoleptic, and chemical–nutritional characteristics of these landraces [27,58,59,60], limited information exists regarding their intra-population variability and genetic relationships with commercial red garlic varieties or other red garlic landraces cultivated in Italy. Specifically, by examining numerous accessions from each landrace, this study aimed to achieve the following objectives: (i) evaluate the genetic and phenotypic intra-population diversity within each landrace; (ii) elucidate the genetic relationships among the accessions of the two landraces and assess their distinctiveness compared to other varieties or landraces cultivated in Italy; (iii) characterize the total phenolic content of the bulbs and investigate its correlation with antioxidant properties.

Although garlic landraces are regarded as distinct yet variable populations, few studies addressing the genetic and phenotypic diversity of garlic have focused on intra-landrace variation [24,56]. Therefore, our research could serve as a case study for describing and quantifying the variability within garlic landraces and for providing an approach to analyzing local adaptation, which is a key factor in their ongoing conservation.

## 2. Materials and Methods

### 2.1. Plant Materials

Thanks to the support of the technicians of ARSIAL, nine accessions of the “Aglio Rosso di Proceno” (codes R_PROC_1–9 in Table S1) and eleven of the “Aglio Rosso di Castelliri” (codes R_CAST_1–11 in Table S1) landraces were collected from farmers in the two traditional areas of cultivation in the 2022 season, prior to sowing the experimental field. The nine and eleven farmers attributed their populations to the “Aglio Rosso di Proceno” and “Aglio Rosso di Castelliri” landraces, respectively, and declared their “seeds” to be historical (grown on the same farm for more than 50 years). Farmers are involved in the “Conservation and Safety Network” coordinated by ARSIAL to maintain in situ landraces for agrobiodiversity conservation.

In the molecular analysis, eight accessions of five Italian red garlic landraces and four commercial varieties were used as reference genotypes (Table S1). The “Aglio Rosso di Sulmona” (codes R_SULM_1–4) is a landrace originating from Sulmona in the province of L’Aquila (Abruzzo, central Italy), which, as previously mentioned, is now cultivated in many regions of central Italy, including the Lazio region, for its valued organoleptic properties and has been enrolled in the “Common catalogue of varieties of vegetable species”. The “Aglio Rosso di Sora” (code R_SORA_1) is a putative landrace grown in the area of Sora in the province of Frosinone (Lazio, central Italy), a small town near the municipality of Castelliri. The “Aglio Rosso Maremmano” (code R_MAREM_1) is a landrace cultivated in the province of Grosseto (Tuscany, central Italy), while the “Aglio Rosso di Cannara” (code R_CANN_1) is a putative landrace grown in the homonymous municipality in the province of Perugia (Umbria, central Italy). The “Aglio Rosso di Nubia” (code R_NUB_1) is a landrace cultivated in the municipality of the same name in the province of Trapani (Sicily, Southern Italy). Two of the four Spanish red garlic commercial varieties (codes R_SPA_1–2) were purchased from local markets in Sulmona (AQ) and Sora (FR), respectively, while the other two varieties, Morado and Gardos (codes R_SPA_3–4), which are widely cultivated in Italy, were supplied by specialized commercial retailers.

Two genotypes were also used as white garlic controls in the molecular analysis (Table S1). One (code B_MONT_1) relates to an accession of a putative landrace cultivated in the coastal areas of upper Lazio and collected from a farm in the municipality of Montalto in the province of Viterbo. The other (code B_LAZIO_SUD) is a Spanish white garlic variety widely cultivated in southern Lazio and purchased from a specialized commercial retailer. Finally, an accession of the “Aglione della Chiana” (code AGLIONE), belonging to the species *Allium ampeloprasum* var. *holmense*, was also used as an outgroup genotype. “Aglione della Chiana” is a landrace cultivated in an area between the provinces of Arezzo, Siena, and Perugia (central Italy), characterized by larger bulbs and a more delicate aroma than common garlic.

### 2.2. DNA Extraction and Molecular Markers Analysis

Genomic DNA was extracted from the garlic bulbs of a single individual plant per accession using the NucleoSpin^®^ Plant II kit (Macherey-Nagel, Düren, Germany), according to the manufacturer’s instructions. A 0.8 (*w*/*v*) agarose gel stained with ethidium bromide (0.001%) and a Nanodrop Bioanalyzer ND1000 (ThermoScientific, Waltham, MA, USA) were used to evaluate the integrity and concentration of DNA. All DNA samples were diluted to 50 ng/µL with distilled water and stored at −20 °C until use.

Thirteen SSR loci [53,54,55] were selected from previous genetic studies of garlic germplasm collections [56,57,58,59,60], based on their high values of PIC (polymorphic information content) (Table S2). SSR primers were multiplexed, by labeling their forward primer with TAMRA, JOE, and FAM fluorescent dyes (Eurofins Genomics, Ebersberg, Germany). PCR reactions were conducted as described previously [61], using the specific annealing temperature of each primer pair (Table S2). Amplification fragments were separated by capillary electrophoresis using an ABI 3130 DNA Analyzer (Applied Biosystems, Waltham, MA, USA). The Gene Mapper 4.0 software was used to determine allele sizes, based on the GeneScan LI500Liz size standard (Applied Biosystems, Waltham, MA, USA).

DNA samples were also amplified with ten ISSR primers from the British Columbia University (UBC) collection (Table S3). Seven of these primers were previously found to be highly informative for studying the genetic relationships between several genotypes of the genus *Platanus* [62,63] and various globe artichoke landraces/clones belonging to the “Romanesco” varietal type [64]. PCR reactions and the analysis of amplification products were carried out in accordance with Alicandri et al. [61].

### 2.3. Field Trial

Only a subset of the genetically characterized garlic accessions was used to set up the experimental field trial, which included the 20 accessions of the “Aglio Rosso di Proceno” and “Aglio Rosso di Castelliri” landraces. Additionally, three accessions of the “Aglio Rosso di Sulmona” landrace (R_SULM_2–4), the accessions of the three landraces “Aglio Rosso di Sora” (R_SORA_1), “Aglio Rosso Maremmano” (R_MAREM_1), and “Aglio Rosso di Cannara” (R_CANN_1), and two Spanish commercial varieties of red garlic (R_SPA_2 and R_SPA_4), were included as control genotypes. The 28 garlic accessions were field grown at the ARSIAL experimental station located in Alvito (Lazio region, province of Frosinone: 41°40′12″ N; 13°44′87″ E; altitude 482 m a.s.l.; average annual temperature 11.4 °C; average annual rainfall 1077 mm). The main characteristics of the soil in the Alvito field where the garlic accessions were cultivated are reported in Table S4.

On 25 November 2022, cloves were sown directly in the field, without any pretreatment, following the traditional agricultural practices in the two locations of origin (Proceno and Castelliri) of the two landraces under study. Cloves were planted in rows separated by 0.7 m, with a distance of 0.2 m between each plant. The plant material was arranged in a randomized block design, with two replications, and each experimental unit (plot) consisted of 30 plants per accession. All 28 accessions were harvested on 13 July 2023 (230 days after sowing). After harvesting, roots and stalks were removed, and bulbs were transported to the laboratory for further analysis.

### 2.4. Morphological Analysis of Bulbs and Cloves

At harvest, four bulbs for each accession (two bulbs for each replicate) were randomly selected from each plot in the experimental field. Pictures of the collected bulbs for morphological analysis from representative accessions of the “Aglio Rosso di Castelliri” and “Aglio Rosso di Proceno” landraces are shown in Figures S1 and S2. Morphological and biometrical measurements on bulbs and cloves were performed by means of 27 traits (10 qualitative and 17 quantitative) (Table S5). Qualitative descriptors were adapted from the International Union for the Protection of New Varieties of Plants (UPOV) [65] and International Plant Genetic Resources Institute (IPGRI) [66] and were the following: shape of mature dry bulbs (SMDB), bulb shape in longitudinal section (BSLS), bulb shape in cross section (BSCS), outer skin color of compound bulb (OSCB), presence of anthocyanins bulb (PAB), bulb distribution of cloves (BDC), bulb external cloves (BECs), bulb structure type (BST), skin color of the clove (SCC), and peeled clove color (PCC).

Nine quantitative traits related to weight and dimensional parameters were measured in the four bulbs per accession: weight (W); equatorial diameter (De); polar diameter (Dp); thickness (T); diameter geometric mean (Dgm); diameter arithmetic mean (Dam); surface area (SA); cross-sectional area (CSA); sphericity index (SI) (Table S5) [67]. Weight was determined using a bench scale (PFB-2000-2, Kern & Sohn GmbH, Ballingen, Germany) with an accuracy of ±0.01 g. Linear dimensions were obtained using a digital caliper (Digital Caliper, Hylka Tools, Chessington, UK) with an accuracy of ±0.1 mm. After counting the number of cloves per bulb (NCLbulb), all cloves were weighted and the average clove weight per bulb (CLW) was determined. Peeled cloves from the four fresh bulbs per accession were weighed and subsequently subjected to drying in an oven set at 60 ± 1 °C until they reached a constant weight. The determination of dry matter content (DM) was conducted by calculating the difference in weight between the fresh and dried samples, expressed as a percentage (%) of the fresh weight.

Measurements of color were assessed for two peeled cloves per bulb using a Minolta colorimeter (Minolta C2500, Konica Minolta, Ramsey, NY, USA) to determine chromaticity values in terms of CIELab color space, L* (lightness), a* (green to red), and b* (blue to yellow). In addition, the derived parameter chroma (C*), indicating, respectively, the vividness/dullness, was evaluated. The hue angle (h) was calculated by chromaticity values a* and b* using a method reported earlier by McGuire [68]. Two readings were taken from the two equatorial faces of each clove.

### 2.5. Total Phenolics and Antioxidant Activity

Out of the 20 accessions of the two landraces, “Aglio Rosso di Castelliri” and “Aglio Rosso di Proceno”, enrolled in the experimental field trial, 13 were selected based on their genetic relationships determined using SSR and ISSR markers for the analyses of polyphenol content and antioxidant activity of their bulbs. Specifically, the selected accessions included R_PROC_3, R_PROC_5, R_PROC_6, R_PROC_7, R_PROC_8, R_PROC_9, R_CAST_1, R_CAST_3, R_CAST_4, R_CAST_5, R_CAST_6, R_CAST_8, and R_CAST_9. Additionally, as control genotypes, two accessions from the “Aglio Rosso di Sulmona” landrace (R_SULM_3–4), two Spanish commercial varieties of red garlic (R_SPA_2 and R_SPA_4), and the single accession from the landrace “Aglio Rosso di Sora” (R_SORA_1) were also included in the analyses.

The preparation of garlic samples to develop sulfur compounds, believed to be key components in the generation of antioxidant compounds in garlic, involved activating the enzyme alliinase through cell rupture, representing the first step in the transformation from inactive forms (e.g., alliin) to bioactive compounds (e.g., di-trimethylsulfides, ajoene). For this purpose, whole garlic bulbs were stripped of their tunics, reduced to cloves, mixed with water at a 1:10 ratio, and crushed inside an impermeable sachet (Whirl-Pak) for 5 min until a mushy consistency was achieved. The mush was then placed in an ultrasonic bath (Argolab Mod. DU-32, ArgoLab, Carpi, Italy) at 26 kHz for 20 min. Afterward, the samples were transferred to 50 mL tubes and centrifuged (Neya 16R) at 8500 rpm for 10 min. The supernatant was collected and stored at −20 °C until analysis. The total phenolic and antioxidant compounds were extracted from ground tissues (1.0 g), homogenized in an 80% ethanol (10 mL) solution, and subsequently centrifuged at 11,200 rpm for 10 min. The total phenolic content was determined as described by Singleton and Rossi [69] with slight modification. Briefly, 120 μL of the extract was mixed with 50 μL of Folin–Ciocalteu reagent and left for 3 min at room temperature. After that, 30 μL of saturated sodium carbonate solution was added and the mixture was incubated at 36 °C for 60 min and read at 765 nm using a UV-Vis spectrophotometer (Perkin Elmer Lambda 850+). Phenolic concentration was calculated by interpolation with a calibration curve (0, 50, 100, 150, 250, and 500 µg/mL) of gallic acid (R^2^ 0.9996) and the results expressed as mg of gallic acid equivalents on a fresh weight basis (mg GAE/100 g fw).

The assessment of antioxidant activity was conducted by the DPPH assay following the methodology outlined by Blois [70] with slight modifications. Two solutions were prepared: one containing 6-hydroxy-2,5,7,8-tetramethylchroman-2-carboxylic acid (Trolox), serving as a positive control, and another containing a 0.2 mM solution of DPPH, used as a negative control. An amount of 100 µL of the extract was mixed with 1.0 mL of a DPPH solution (3.9 mg DPPH dissolved in 100 mL of methanol/water in a ratio of 80:20 *v*/*v*). This mixture was left to incubate for 1 h in darkness at room temperature. Following incubation, the absorbance was measured at 517 nm using a spectrophotometer (Perkin Elmer Lambda 850+). The DPPH radical scavenging activity was presented as a function of the concentration of Trolox equivalent antioxidant capacity (TEAC) and expressed as mM of Trolox equivalents on a fresh weight basis (mM TE/g fw).

For each biological replicate (four bulbs), two measurements (analytical replicates) were obtained to determine the total phenolic content and antioxidant activity.

### 2.6. Data Analysis

#### 2.6.1. Molecular Data

For SSR markers, each allele was recorded based on its size (pb) and their screening power was assessed by calculating the PIC index [71], using Power Marker 3.25 [72]. The following parameters were used to assess genetic diversity per locus: number of observed alleles per locus (Na), number of private alleles, major allele frequency (MAF), and observed and expected heterozygosity (Ho and He, respectively), using GenAlEx6 [73] and Power Marker 3.25 [72].

The DNA fragments in all genotypes were scored as present (1) or absent (0) for each ISSR primer and the raw data were imported into a Microsoft EXCEL spreadsheet to generate a binary matrix. The genetic diversity of each ISSR primer was evaluated using the parameters listed below: number of total bands (NTB) produced by each ISSR primer, number of polymorphic bands (NPB), percentage of polymorphism (% Pol), number of private alleles, MAF, and He, with the last two parameters calculated using Power Marker 3.25 [72]. The PIC values of the ISSR primers were estimated by using the equation proposed by Serrote et al. [74]: PIC = 1 − (p^2^ + q^2^), where p and q indicate the frequency of bands present or absent in the individuals in a population, respectively.

A single matrix was produced for the combined analysis of SSR and ISSR markers by converting the sizes of the SSR amplification fragments into 1/0 values (presence/absence). The coefficient of Nei [75] was used to estimate the genetic distances for phylogenetic relationships between the different accessions. The obtained distances matrix was employed to construct a phylogenetic tree using the UPGMA clustering method in MEGAX software version 10.2.4 [76]. The reliability of the tree topology was evaluated using bootstrapping over 1000 replicates with the PAUP* 4.0 software [77]. In addition to the UPGMA clustering analysis, a principal coordinate analysis (PCoA) was performed using GenAlEx6 version 6.5 [73], and the first two principal coordinates were plotted in two-dimensional space.

To evaluate the genetic structure of the garlic accessions, a Bayesian-based clustering method was applied on multi-locus SSR and ISSR data using STRUCTURE v. 2.3.4 software [78]. To determine the optimal number of clusters (K), an admixture model was employed alongside correlated allele frequencies, utilizing a burn-in period of 50,000 and 500,000 Markov Chain Monte Carlo (MCMC) simulations. The range of tested clusters (K) spanned from 1 to 10, with 10 iterations conducted for each simulated K value. The estimation of the number of clusters employed two distinct methodologies. Firstly, the ΔK method proposed by Evanno et al. [79] was applied to ascertain the most probable number of clusters (K), using STRUCTURE HARVESTER version 0.6.94 [80], and selecting the optimal K value corresponding to the highest ΔK. Accessions with a probability of membership (Q) value > 0.75 were assigned to their corresponding genetic clusters. Secondly, the post hoc approach proposed by Pritchard et al. [81] was employed to detect substructures or “nested” clusters after the first STRUCTURE analysis. For this, a second round of clustering by STRUCTURE of the clusters obtained in the first analysis was performed, and the accessions were assigned to a cluster if their Q value was higher than 0.75.

The genetic diversity of the “Aglio Rosso di Castelliri” and “Aglio Rosso di Proceno” landraces was assessed by calculating Ne, He, the number of private alleles, and the Shannonver information index (I) for both the ISSR and SSR markers using GeneAlEx6 [73] and Power Marker 3.25 [72]. In addition, the observed heterozygosity (Ho) was also calculated for SSR markers in GeneAlEx6 [73]. To test the significance of the differences in the genetic parameters between the two landraces the Kruskal–Wallis nonparametric test was performed using JMP PRO 15 (©SAS Institute Inc., Cary, NC, USA). The analysis of molecular variance (AMOVA) between and within landraces was performed using a distance matrix and suppressing within the individual analysis, as defined in GenAlEx6 [73]. The variance components were tested statistically by nonparametric randomization tests using 9999 permutations. Finally, the fixation index Fst [82] was calculated to assess the level of genetic differentiation among the different Castelliri and Proceno garlic populations using GenAlEx 6 software [73] for the codominant markers and PopGene software (version 1.32) [83] for the dominant markers.

#### 2.6.2. Morphological and Color Data

Morphological and color data of bulbs and cloves were analyzed using one-way ANOVA, principal component analysis (PCoA), and cluster analysis. The means were compared by using Tukey’s pairwise tests at a significance level of *p* < 0.05. The Shapiro–Wilk test was used to assess the normality of distribution of the observations. The coefficient of variation (i.e., the value of the standard deviation of the mean divided by the mean), expressed as a percentage, was used to analyze the variability of the morphological traits between the analyzed accessions. Furthermore, Pearson correlation coefficients were calculated to identify possible correlations between morphological traits. Based on the morphological and color data, a similarity dendrogram was constructed using hierarchical cluster analysis. All these statistical analyses were performed using JMP PRO 15 (©SAS Institute Inc., Cary, NC, USA). The correlation between the cophenetic matrix of Euclidean distances (morphological and color data) and the cophenetic matrix of genetic distances based on SSR and ISSR data among the 28 garlic accessions was tested using the Mantel test in GeneAlEx6 [73]. *p*-values were calculated using 9999 permutations. ANOVA, followed by Tukey’s pairwise tests at a significance level of *p* < 0.05, was used to compare the morphological and color data of the accessions belonging to the different clusters identified by cluster and PCA analyses.

#### 2.6.3. Phenolic and Antioxidant Data

Data of the total phenolic content and antioxidant properties of bulbs were submitted to one-way ANOVA by using JMP PRO 15 (©SAS Institute Inc., Cary, NC, USA), and the means were compared by using Tukey’s pairwise tests at a significance level of *p* < 0.05. The coefficient of variation was used to analyze the variability of the total phenolic and antioxidant data between the analyzed accessions. The correlation between the cophenetic matrix of Euclidean distances (total phenolics and antioxidant activity) and the cophenetic matrix of genetic distances based on SSR and ISSR data among 18 selected garlic accessions was tested using the Mantel test in GeneAlEx6 [73]. In addition, JMP PRO 15 (©SAS Institute Inc., Cary, NC, USA) was used for analyzing the correlation between the total phenolic content and antioxidant activity of bulbs.

## 3. Results

### 3.1. Genetic Analysis by SSR and ISSR Markers

#### 3.1.1. SSR and ISSR Markers Polymorphism and Their Informativeness

All the SSR markers tested were polymorphic in all the accessions analyzed and their main genetic parameters are reported in Table 1. In the entire collection, which includes the 34 accessions of *A. sativum* and the accession of the landrace “AGLIONE” of *A. ampelosarum* var. *holmense*, a total of 68 alleles were identified for the codominant SSR markers. The number of alleles ranged from four (GB_ASM_059, Asa_17, and AS_589) to seven (AS_987 and AS_5944), with an average of 5.23 alleles per locus (Table 1). In contrast, in the 34 garlic accessions, the total number of alleles identified was 57, with an average of 4.38 alleles per locus. The number of alleles ranged from three (GB_ASM_059, Asa_24, and AS_30) to six (AS_11065 and AS-987). Six accessions belonging to six different landraces/commercial varieties showed 22 private alleles, with the accession “AGLIONE”, belonging to the species *Allium ampeloprasum* var. *holmense*, holding the highest number, corresponding to 11 private alleles at 10 different loci, followed by the red garlic landrace R_NUB_1, with four private alleles at three different loci (Table S6).

In the entire collection, the expected heterozygosity (He) ranged from 0.14 (AS_30) to 0.73 (AS_739), with a mean of 0.50, while the observed heterozygosity varied from 0.03 (Asa_17 and AS_589) to 1 (GB_ASM_078 and GB_ASM_059), with a mean of 0.55 (Table 1). In the 34 garlic accessions, He varied from 0.09 (AS_30) to 0.74 (AS_739), with an average of 0.48, while Ho ranged from 0.03 (Asa_17 and AS_589) to 1 (GB_ASM_078 and GB_ASM_059), with a mean of 0.46.

The markers’ discriminatory power, measured by the polymorphic information content (PIC), varied from 0.16 to 0.60, with an average of 0.41 in the entire collection, while in the 34 garlic accessions, PIC values ranged from 0.11 to 0.57, with a mean of 0.38. Overall, the most informative SSR loci were AS_5944, AS_987, AS_11065, and AS_739, which showed the highest PIC values (0.54–0.60 in the entire collection; 0.51–0.57 in the 34 garlic accessions) and He values (0.56–0.73 in the entire collection; 0.54–0.74 in the 34 garlic accessions), and among the lowest MAF values (Table 1). Importantly, these loci are among those with the highest number of alleles (Table 1). Based on the calculated genetic parameters, GB_ASM_040, Asa_24, and AS_589 can also be considered good markers with the PIC values ranging between 0.47 and 0.51 in the entire collection, and between 0.44 and 0.48 in the 34 garlic accessions (Table 1).

Based on the ISSR analysis, a total of 181 clear and reproducible bands, ranging from 300 bp to 3 Kb in size, were generated across the all the accessions analyzed, with an average of 18.1 bands per primer (Table 1). As an example, Figure S3 shows the polymorphic patterns of four out of 10 ISSR primers from representative accessions of “Aglio Rosso di Proceno” and “Aglio Rosso di Castelliri”, along with garlic landraces/varieties used as references. In the entire collection, the number of bands varied from 14 (primer UBC_860) to 22 (primers UBC_851 and UBC_881), with all bands found to be polymorphic among the analyzed garlic accessions. In contrast, 146 clear and reproducible ISSR bands were identified in the 34 garlic accessions, with an average of 14.6 bands per primer (Table 1). The number of bands ranged from 11 (primer UBC_860) to 20 (primer UBC_851). Moreover, UBC_860 and UBC_851 produced the lowest (eight) and the highest (20) number of polymorphic bands, respectively (Table 1). Overall, 131 polymorphic bands were detected, representing 89% polymorphism, in the 34 garlic accessions.

Approximately 31% (56/181) of bands (alleles) were private, with the highest number (32) found for the accession “AGLIONE”, followed by the two white garlic accessions B_LAZIO_SUD (16) and B_MONT_1 (six) (Table S7).

In the entire collection, He and PIC values for the ten ISSR primers ranged from 0.08 (UBC_881) to 0.30 (UBC_848) with mean values of 0.17 (Table 1). In the 34 garlic accessions, the PIC and He values ranged from 0.09 (UBC_881 and UBC 860) to 0.33 (UBC_848), with an average of 0.18 (Table 1).

#### 3.1.2. Genetic Relationship Among Garlic Accessions

**Figure 1:**
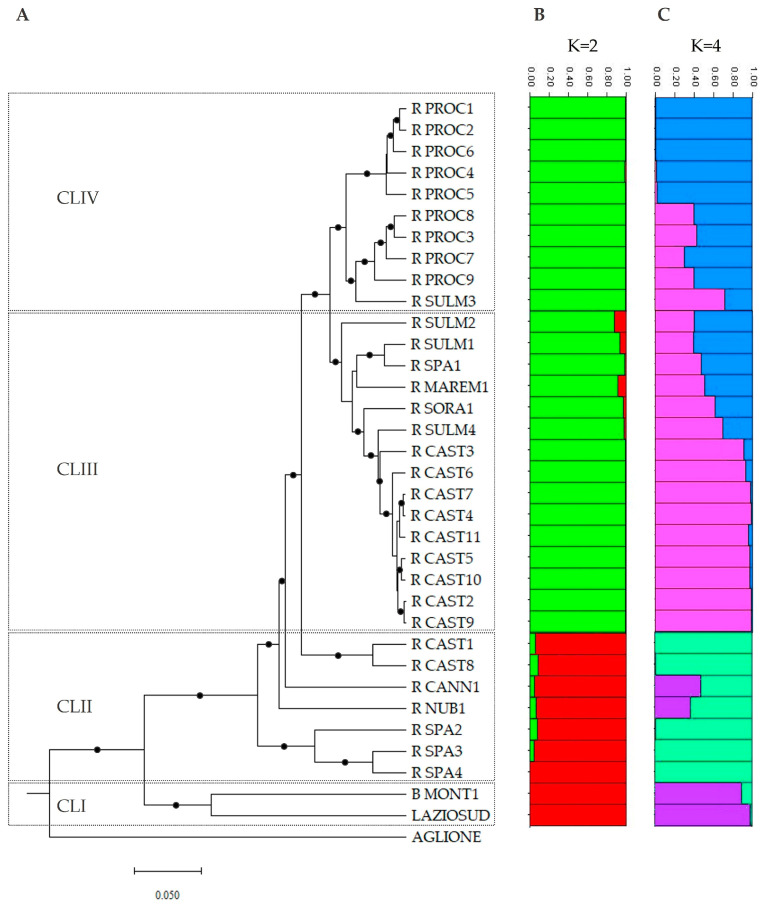
Cluster analysis of the 34 *A. sativum* accessions and the outgroup species *A. ampeloprasum* var. *holmense* (AGLIONE). (**A**) UPGMA tree based on Nei’s coefficient [75] among the 34 *A. sativum* accessions and the outgroup species. Clusters I–IV indicate the groups revealed by clustering the 34 *A. sativum* accessions. Branches indicated with dots represent bootstrap support of more than 80% (1000 repetitions). Structure bar plots of average proportion (q) for the 34 *A. sativum* accessions for K = 2 (in green and red) obtained following the first STRUCTURE analysis (**B**) and for K = 4 (in pink, cerulean, light green, and purple) obtained from the second STRUCTURE analysis (**C**).

The UPGMA dendrogram based on the genetic distances between the 35 garlic accessions, estimated by Nei’s coefficient [75] using both SSR and ISSR molecular markers, is reported in Figure 1A. The outgroup genotype AGLIONE, belonging to the species *A. ampeloprasum* var. *holmense*, was distinctly separated from all the *A. sativum* accessions forming the basal branch of the tree and demonstrating a coherent genetic clustering of the taxa. The 34 accessions of *A. sativum* can be divided into four main clusters (CLI–IV in Figure 1A). CLI included the two accessions of white garlic used as controls, which were clearly distinguishable from all the red-type garlic accessions. CLII, on the other hand, comprised three out of the four commercial varieties of Spanish red garlic (R_SPA_2, -3, and -4), along with two accessions of the landrace “Aglio Rosso di Castelliri” (R_CAST_1 and -8), as well as two accessions related to the landraces “Aglio Rosso di Nubia” (R_NUB_1) and “Aglio Rosso di Cannara” (R_CANN_1). CLIII included nine out of 11 accessions of the landrace “Aglio Rosso di Castelliri”, three accessions of the landrace “Aglio Rosso di Sulmona” (R_SULM_1, -2, and -4), the two accessions related to the landraces “Aglio Rosso Maremmano” and “Aglio Rosso di Sora” (R_MAREM_1 and R_SORA_1), and the putative Spanish commercial variety R_SPA_1. Finally, cluster IV comprised the nine accessions of the landrace “Aglio Rosso di Proceno” and one of the four accessions of the landrace “Aglio Rosso di Sulmona” (R_SULM_3). It is interesting to note that the nine accessions of the landrace “Aglio Rosso di Castelliri” were grouped together in cluster III, depicting a relatively high level of genetic similarity among them. In contrast, the nine accessions of the landrace “Aglio Rosso di Proceno” in cluster IV were divided into two genetic groups, consisting of five and four distinct accessions, respectively, with the accession R_SULM_3 grouping with the latter (Figure 1A).

PCoA was performed to corroborate the results obtained by the UPGMA clustering analysis (Figure S4). In the PCoA, the first three coordinates explained 54.73% of the total genetic variation among the 35 garlic accessions, with the first accounting for 27.29%, the second for 15.43%, and the third for 12.01%. The PCoA results were largely congruent with those obtained from the UPGMA analysis. Indeed, the outgroup accession AGLIONE and the two white garlic accessions were clearly separated from each other and from all the red-type garlic accessions (Figure S4). Among the 32 red garlic accessions, those assigned to clusters III and IV following UPGMA analysis, including, among others, nine of the 11 accessions of the landrace “Aglio Rosso di Castelliri” and the nine of the landrace “Aglio Rosso di Proceno”, respectively, formed two distinct groups placed on the lower left and upper left parts of the graph. On the other hand, the seven accessions assigned to cluster II by UPGMA analysis were placed between these two groups and the two white garlic accessions (Figure S4). According to the UPGMA analysis, the nine accessions of the landrace “Aglio Rosso di Proceno” were divided into two genetic groups, comprising five and four distinct accessions, respectively, with the accession R_SULM_3 positioned intermediate to these groups (Figure S4).

#### 3.1.3. Population Structure of Garlic Accessions

A Bayesian-based clustering approach was applied to multi-locus SSR and ISSR data using STRUCTURE software version 2.3.4 to analyze the genetic structure of the garlic collection under study. This method aims to ascertain the optimal number of genetic groups present in the dataset, assign the accessions to each of the identified groups, and identify individuals exhibiting genetic admixture. The analysis exclusively used SSR and ISSR data from the 34 *A. sativum* accessions categorized into the four clusters (I, II, III, and IV) identified by the UPGMA clustering method (Figure 1A).

The first round of STRUCTURE analysis revealed that the garlic collection under study could be represented by two (K = 2) genetic groups (Figure S5A). The first group (in red) included the two white-type garlic accessions, and the seven red-type garlic accessions assigned to CLI and CLII following UPGMA analysis, respectively (Figure 1B). In contrast, the second group comprised 16 and 10 red-type garlic accessions categorized in CLIII and CLIV by UPGMA analysis, respectively (Figure 1B). All genotypes were uniquely assigned to one of the two groups based on the membership probability Q value, which was in all cases > 0.75 for individuals assigned to each of the two genetic groups (Table S8). However, a second round of STRUCTURE analysis for the accessions from the two clusters identified in the first STRUCTURE analysis revealed further sub-structuring or nested clusters, resulting in the subdivision of each cluster into two additional subgroups (Figure S5B,C), totalling four subpopulations (Figure 1C). The four clusters identified following the second STRUCTURE analysis were generally congruent with the clustering results from UPGMA and PCoA analyses, as depicted in Figure 1A,C and Figure S4, and in some cases, validated the correct assignment of accessions to the corresponding landrace or commercial variety.

The first genetic group (in purple in Figure 1C) included the two accessions of white garlic assigned to CLI by UPGMA analysis. The second group (in light green in Figure 1C) comprised the three red-type Spanish commercial varieties and the two accessions of the landrace “Aglio Rosso di Castelliri” (R_CAST_1 and -8) categorized in CLII following UPGMA analysis. Meanwhile, the other two accessions of red garlic (R_NUB_1 and R_CANN_1) assigned to the same cluster were of mixed origin, likely resulting from hybrids between genotypes assigned to the first and second groups (Figure 1C and Table S8). These results suggested that the two accessions R_CAST_1 and -8, due to their genetic similarity to the Spanish red-type commercial varieties, could not be considered to belong to the landrace “Aglio Rosso di Castelliri”.

The third group (in pink in Figure 1C) included the remaining nine accessions of the landrace “Aglio Rosso di Castelliri” assigned to CLIII by UPGMA analysis, while the fourth group (in cerulean in Figure 1C) comprised five of the nine accessions of the landrace “Aglio Rosso di Proceno” that formed a distinct genetic group within CLIV. On the other hand, six red-type garlic accessions used as controls assigned to CLIII, and four accessions of the landrace “Aglio Rosso di Proceno”, which, together with the accession R_SULM_3, formed the second distinct genetic group inside CLIV, could be considered of mixed origin between the third and fourth genetic pools (Figure 1C and Table S8). These results, together with those obtained from the first round of STRUCTURE analysis, suggested a likely common origin of all the accessions assigned to CLIII and CLIV.

It is important to note that the clustering results from UPGMA, PCoA, and STRUCTURE analyses showed that the accession R_SPA_1, classified as a Spanish commercial variety of red garlic, was distinctly different from the other three commercial Spanish red garlic varieties used as controls. In contrast, it was closely genetically associated with three of the four accessions of the landrace “Aglio Rosso di Sulmona” (Figure 1A,C and Figure S4). This accession was purchased from a local vendor in the municipality of Sulmona (see Section 2.1 of Materials and Methods), indicating that it is likely a misclassification, probably being an accession of the landrace “Aglio Rosso di Sulmona” rather than a Spanish commercial variety.

#### 3.1.4. Genetic Diversity Among and Within “Aglio Rosso di Castelliri” and “Aglio Rosso di Proceno” Landraces

Based on the nomenclature assigned to each accession by the farmers and the results of molecular analyses, we categorized nine accessions under each of the two garlic landraces under investigation. This decision was informed by the consideration that two out of the 11 accessions of the landrace “Aglio Rosso di Castelliri” (R_CAST_1 and R_CAST_8) may represent cases of contamination with phenotypically similar foreign material, which, based on the clustering results, is likely genetically associated with commercial varieties of Spanish red garlic.

The diversity analysis of the two landraces, using SSR markers, revealed that “Aglio Rosso di Proceno” exhibited higher values for the effective number of alleles (Ne), Shannon index (I), and expected and observed heterozygosity (He and Ho, respectively) than “Aglio Rosso di Castelliri”, but the differences were not statistically significant according to the Kruskal–Wallis test (Table 2). Of the 31 total alleles detected across the 13 SSR loci in the 18 accessions, nine were specific to one of the two landraces, with “Aglio Rosso di Castelliri” and “Aglio Rosso di Proceno” showing four and five private alleles, respectively (Table 2 and Table S9).

For the ISSR-dominant markers, “Aglio Rosso di Proceno” exhibited significantly higher values of Ne, I, and He compared to “Aglio Rosso di Castelliri”, along with the presence of nine private alleles (Table 2 and Table S10). Interestingly, these nine ISSR private alleles were specific to either individual accessions or groups of accessions within one of the two genetic groups identified in the “Aglio Rosso di Proceno” landrace (Table S10). These ISSR alleles could prove highly beneficial for future clonal selection programs within the “Aglio Rosso di Proceno” landrace, potentially facilitating the development of a simple PCR-based assay for clone identification.

AMOVA analysis showed different results for the two types of molecular markers. For co-dominant SSR markers, the majority of the variability (78%) was attributed to differences among populations (landraces), whereas for dominant ISSR markers, only 45% of the total variation was among populations, with the largest portion (55%) attributed to differences within them (Table 3). These results indicate that SSR markers primarily distinguish accessions between the two landraces, while ISSR-derived polymorphic bands provide better resolution of genetic variability within the two landraces, highlighting the specific usefulness and complementarity of the two marker types used. In addition, the Fst values detected for the two types of molecular markers (0.227 for SSRs and 0.345 for ISSRs in Table 3) revealed a high level of genetic differentiation among the 18 accessions assigned to the two landraces. According to Wright [84], genetic differentiation is classified low for Fst < 0.05, moderate for Fst between 0.05 and 0.15, high for Fst between 0.15 and 0.25, and very high for Fst > 0.25.

### 3.2. Phenotypic Diversity of Bulbs and Cloves Among and Within the “Aglio Rosso di Castelliri” and “Aglio Rosso di Proceno” Landraces, and Comparison with Red-Type Garlic Reference Genotypes

The morphological characterization of the bulbs and cloves was carried out by randomly selecting two plants from each of the two replicates in the experimental field for the eleven accessions of “Aglio Rosso di Castelliri”, the nine accessions of “Aglio Rosso di Proceno”, the six accessions of red-type garlic landraces cultivated in central Italy, and the two Spanish red garlic commercial varieties. The last two groups were used as controls.

#### 3.2.1. Variation in Qualitative Traits Among Garlic Accessions

No substantial differences were found in the ten qualitative descriptors of bulbs among the 28 red garlic accessions analyzed (Table S11). In other words, we did not find any single qualitative descriptor that could discriminate between the “Aglio Rosso di Castelliri” and “Aglio Rosso di Proceno” landraces, or between them and the garlic red-type controls. Overall, the accessions of the two red garlic landraces from the Lazio region produce bulbs wrapped in white tunics with light violet streaks due to a slight presence of anthocyanins. These bulbs have a generally wide elliptical shape in longitudinal section, while in cross-section they are circular. The cloves, varying in shades of violet, are distributed radially and regularly in two sections without the presence of external cloves. The flesh color of the cloves is yellowish.

#### 3.2.2. Variation in Quantitative Weight and Dimensional Traits Among Garlic Accessions

In contrast to qualitative traits, significantly high diversity across the 28 analyzed red garlic accessions was found in 11 out of the 12 quantitative dimensional traits used for the morphological characterization of bulbs and cloves (Table S12). Indeed, ANOVA analysis showed no significant differences among the garlic accessions only for the bulb sphericity index (SI) trait (Table S12). In accordance with the coefficient of variation (CV), the studied red garlic collection exhibited greater phenotypic variability for bulb weight (W, 20.45%), bulb cross-sectional area (CSA, 17.38%), bulb surface area (SA, 16.50%), clove average weight (CLV, 14.02%), and the number of cloves per bulb (NCLBulb, 13.26%) (Table S12). In contrast, relatively lower variability was detected for the remaining seven traits, with CVs ranging between 2.63% and 9.84% (Table S12).

The garlic bulbs of the 28 garlic accessions weighed between 16.28 and 30.30 g, with an overall mean of 21 g (Table S12). They contained a number of cloves ranging from 9.25 to 12.75 g (mean of 10.72 g), with an average weight ranging from 1.56 to 2.38 g and an overall mean of 1.95 g (Table S12). Among the bulb linear dimensions measured, the equatorial diameter (De), polar diameter (Dp), and thickness (T) of garlic accessions ranged from 34.90 to 44.80, 28.39 to 36.15, and 30.60 to 39.78 mm, respectively, with overall means of 38.47, 31.49, and 34.28 mm, respectively (Table S12). On the other hand, the geometric mean diameter (Dgm) and arithmetic mean diameter (Dam) of garlic bulb accessions ranged from 30.56 to 38.65 and 31.77 to 40.27 mm, respectively, with overall means of 33.42 and 34.75 mm, respectively (Table S12). Based on the linear dimensions measured, we calculated the surface and cross-sectional areas (SAs and CSAs, respectively), and the shape index (SI) of bulbs. These are considered useful physical parameters for developing new processing equipment and control strategies for storage [67,85]. In particular, SA and CSA are useful for estimating the garlic respiration rate during storage, determining heat transfer [67], and the requirement of chemicals during the application of lye peeling [85]. SI is very important for designing mechanical sorters and graders, and it is also helpful in designing the mechanical peeler for efficient peeling [67]. The SAs and CSAs of garlic bulb accessions ranged from 807.80 to 1276.04 and 2384.20 to 3824.27 mm^2^, respectively, with overall means of 957.06 and 2864.83 mm^2^ (Table S12). The bulb SI ranged from 1.13 to 1.22, indicating that all the bulbs of the garlic accessions studied were spherical in shape according to Abd-Alla [86]. It is interesting to note that, except for SI, the landrace accession “Aglio Rosso di Cannara” (R_CANN_1) exhibited the highest values for all the bulb dimensional traits measured, including the number of cloves per bulb and their average weight, while the lowest values were detected in some accessions assigned to the “Aglio Rosso di Proceno” and “Aglio Rosso di Castelliri” landraces (Table S12). Finally, the bulb dry matter of garlic accessions ranged from 35.99% to 46.38%, with an overall mean of 42.57% (Table S12). The highest value was observed in one of the accessions (R_CAST_7) assigned to the “Aglio Rosso di Castelliri” landrace, while the lowest value was found in the landrace accession “Aglio Rosso di Cannara” (R_CANN_1) (Table S12).

According to the calculated correlation coefficients, bulb weight (W) had a very strong positive correlation with all the measured bulb dimensional traits, the number of cloves per bulb (NCLBulb), and their average weight (CLV) (Table S13). Additionally, all the dimensional bulb traits showed a strong positive correlation with each other. However, NCLBulb had only a weak, although significant, correlation with CLV. Conversely, bulb weight and all the bulb dimensional traits showed a strong negative correlation with bulb dry matter (DM) (Table S13).

#### 3.2.3. Variation in Flesh Clove Color Parameters Among Garlic Landraces

The color of flesh cloves in garlic is considered an important sensory indicator with strong association with texture, taste, appearance, flavor, and overall impression [87]. In our study, the color of peeled cloves was measured using the L* (lightness), a* (red–green component) and b* (yellow–blue component) color coordinates. Hue and chroma were also considered. All garlic accessions were characterized by a pronounced yellow component in their clove flesh (positive b* coordinate) and a minimal greenish component (slightly negative a* coordinate) (Table S14). Consequently, the resulting chroma (C*) and hue (H) values identified the color as yellowish, as confirmed through visual inspection. However, significant differences were observed in the intensity of lightness (L*), yellow (b*) and greenish (a*) tonalities, as well as in C* and hue (H) values among the peeled cloves of the 28 garlic accessions (Table S14). According to the coefficient of variation (CV), the red garlic collection showed the greatest variability for the a* coordinate (CV = 25.03%), followed by the b* coordinate and chroma (CV= 9.94 and 9.93%, respectively) as well as the L* coordinate (CV = 4.67%). The lowest level of variability was detected for the hue parameter (CV = 0.89%) (Table S14).

The L* coordinate of peeled cloves from the garlic accessions ranged from 65.84 in the landrace accession R_CANN_1 to 79.22 in the landrace accession R_MAREM_1, with an overall mean of 73.86 (Table S14). On the other hand, the a* coordinate ranged from –1.96 in the landrace accession R_SULM_4 to −0.80 in the landrace accession R_CANN_1, with a mean of −1.42. The b* and C* values ranged from 20.59 to 27.18 and from 20.63 to 27.25, respectively, with overall means of 23.30 and 23.35, respectively (Table S14). The highest values for the two-color parameters were observed in the accession R_CAST_3, which is assigned to the “Aglio Rosso di Castelliri” landrace, while the lowest values were found in the accession R_PROC_5, which belongs to the “Aglio Rosso di Proceno landrace (Table S14). Finally, the hue values ranged from 92.02 in the landrace accession R_CANN_1 to 95.00 in the landrace accession R_SULM_2, with an overall mean of 93.50 (Table S14).

#### 3.2.4. Cluster and Principal Component Analysis

**Figure 2:**
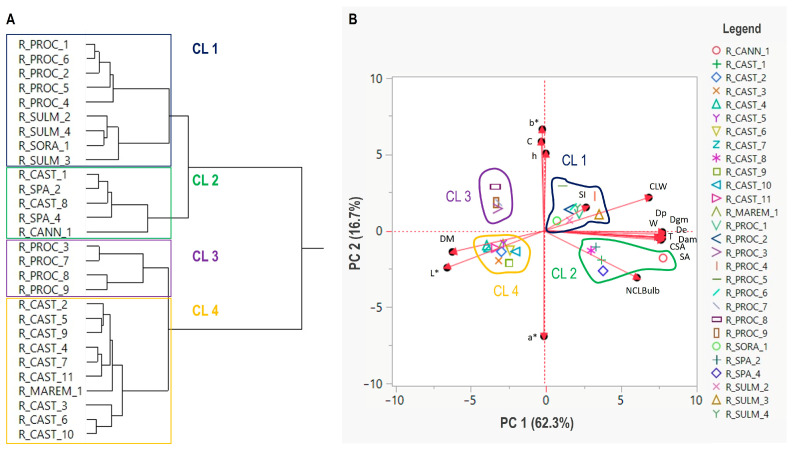
Cluster and principal component analysis based on morphological quantitative traits of the 28 garlic accessions. (**A**) Cluster dendrogram of garlic accessions based on 12 morphological traits of bulbs and cloves, along with five flesh clove color parameters. (**B**) Principal component analysis bi-plot (PC1 vs. PC2) showing the spatial distribution of the morphological and color traits as influenced by garlic accessions. Refer to Table S5 for acronyms of quantitative traits. Four clusters (CLI–IV) indicated by blue, green, purple, and yellow colors, respectively, were identified by both analyses. These clusters included nine, five, four, and ten accessions, respectively.

Using morphological and color data, a similarity dendrogram was generated through hierarchical cluster analysis to illustrate the relationships among the garlic accessions. The phenotypic-based dendrogram revealed that the 28 red garlic accessions could be grouped into four major clusters (CLI–IV in Figure 2A). CLI included five of the nine accessions of the “Aglio Rosso di Proceno” landrace (R_PROC_1–2 and R_PROC_4–6), the three accessions of the “Aglio Rosso di Sulmona” (R_SULM_2–4), and the landrace accession R_SORA_1. CLII comprised two of the 11 accessions of the “Aglio Rosso di Castelliri” landrace (R_CAST_1 and R_CAST_8), which clustered together with the two Spanish red garlic commercial varieties (R_SPA_2 and R_SPA_4) and the landrace accession R_CANN_1. CLIII consisted of the remaining four accessions of the “Aglio Rosso di Proceno” landrace (R_PROC_3 and R_PROC_7–9), while the remaining nine accessions of the “Aglio Rosso di Castelliri” landrace grouped together with the landrace accession R_MAREM_1 to form CLIV (Figure 1A).

PCA was conducted to validate the findings from the hierarchical clustering analysis and to examine the correlations between the morphological and color traits of the bulbs and cloves in the 28 red garlic accessions (Figure 2B). The first two principal components (PCs) had eigenvalues higher than two and together accounted for about 79% of the total variance (Table S15 and Figure 2B). PC1 (62.3% of variance) included contributions for all the bulb dimensional traits (De, Dp, T, Dgm, Dam, SA, CSA, and SI), bulb weight (W), number of cloves per bulb (NCLBulb) and their average weight (CLW), dry matter (DM), and lightness of peeled cloves (L*) (Table S15 and Figure 2B). PC2 explained 16.7% of variance and was correlated with the remaining four flesh clove color parameters (a*, b*, C, and h) (Table S15 and Figure 2B). The PCA results were fully concordant with those obtained from the clustering analysis. The distribution of the garlic accessions against the first two discriminant functions showed that four groups could be identified (Figure 2B), corresponding precisely to the four clusters (CL1–IV in Figure 2A) obtained from the hierarchical clustering analysis.

Possible associations between morphological and color data and the genetic groupings of the 28 garlic accessions based on SSR and ISSR markers data were investigated using the Mantel test (Table 4). The results revealed a moderate and significant overall correspondence between the genetic clustering of the accessions and all the investigated morphological and color traits of bulbs and cloves (r = 0.51, *p* < 0.001). A similar analysis considering some major phenotypic variables separately revealed moderate and significant correlations (r ranging from 0.38 to 0.45, *p* < 0.001) between the genetic clustering of accessions and the weight (W), dimensional parameters (De, Dp, and T), and dry matter (DM) of bulbs, as well as the number of cloves per bulb (NCLBulb). However, no significant association was found for the clove average weight (CLW) (r = 0.18, *p* = 0.08). Among the color parameters of peeled cloves, significant but weak correlations were detected between genetic clustering and the coordinates L* (r = 0.29, *p* = 0.013) and a* (r = 0.28, *p* = 0.002), while no significant association was found for the coordinate b* (r = 0.18, *p* = 0.08).

The potential differences in the major morphological and color traits of bulbs and cloves among the accessions belonging to the four clusters identified by cluster and PCoA analyses (CLI–IV in Figure 2A,B) were further examined (Table 5). The results showed that the accessions within the CLI and CLII clusters were characterized by larger bulb sizes, with all the dimensional bulb traits, bulbs and clove weights, and the number of cloves per bulb significantly higher compared to the accessions in the CLIII and CLIV clusters. In contrast, the CLI and CLII accessions exhibited lower values of dry matter content compared to the accessions in the CLIII and CLIV clusters. Regarding the flesh clove color profile, the CLII and CLIV accessions showed significantly higher values for the a* coordinate than the accessions in the CLI and CLIII clusters, while the b* coordinate values were significantly lower in the CLII and CLIV accessions compared to the CLI and CLIII accessions. Finally, the CLIV accessions exhibited significantly higher values for the L* coordinate compared to the accessions in the CLI, CLII, and CLIII clusters.

### 3.3. Variation in Total Phenolics and Antioxidant Activity of Bulbs Among and Within “Aglio Rosso di Castelliri” and “Aglio Rosso di Proceno” Landraces, Compared to Red-Type Garlic Reference Genotypes

Out of the 20 accessions of the “Aglio Rosso di Castelliri” and “Aglio Rosso di Proceno” landraces included in the experimental field trial, 13 were selected for analysis of polyphenol content and antioxidant activity based on their genetic relationships as determined by SSR and ISSR markers. The selected accessions for the “Aglio Rosso di Proceno” landrace were R_PROC_3, R_PROC_5, R_PROC_6, R_PROC_7, R_PROC_8, and R_PROC_9 (see Figure 1 for their genetic distinctiveness). For the “Aglio Rosso di Castelliri” landrace, the selected accessions were R_CAST_3, R_CAST_4, R_CAST_5, R_CAST_6, R_CAST_9, as well as R_CAST_1 and R_CAST_8, which, based on genetic analyses, can be considered outliers for this landrace (Figure 1). Additionally, for comparative purposes, the analyses of total phenolics and antioxidant activity included two accessions from the “Aglio Rosso di Sulmona” landrace (R_SULM_3 and R_SULM_4), two Spanish commercial varieties of red garlic (R_SPA_2 and R_SPA_4), and one accession from the “Aglio Rosso di Sora” landrace (R_SORA_1).

Significant variation (*p* < 0.001) was found in the bulb concentration of total phenolics and their antioxidant activity among the 18 red garlic accessions (Table 6), with a strong positive correlation between them (r = 0.90, *p* < 0.01). According to the coefficient of variation (CV), the antioxidant activity showed higher variability (CV = 39.67%) than the total phenolics (CV = 24.07%). The bulb total phenolic content of garlic accessions ranged from 33.02 to 76.24 mg GAE/100 g fw, with a mean of 42.59 mg GAE/100 g fw (Table 6). The antioxidant activity of bulbs ranged from 1.96 to 7.81 mM TE/g fw, with a mean of 4.24 mM TE/g fw (Table 6). The highest values for both the total phenolics and antioxidant activity were observed in two accessions of the “Aglio Rosso di Proceno” landrace (R_PROC_5 and R_PROC_8), while the lowest values were found in the two outliers’ accessions, R_CAST_1 and R_CAST_8, of the “Aglio Rosso di Castelliri” landrace (Table 6).

The Mantel test revealed no significant correlations between the genetic clustering of the 18 accessions and their bulb total phenolics and antioxidant activity (Table S16).

Finally, for comparative purposes, we examined the bulb content of total phenolics and their antioxidant activity across three groups of garlic accessions. The first group comprised six accessions assigned to the “Aglio Rosso di Proceno” landrace. The second group consisted of five accessions assigned to the “Aglio Rosso di Castelliri” landrace. The third group included seven accessions used as control genotypes, which encompassed the three accessions from the “Aglio Rosso di Sulmona” and “Aglio Rosso di Sora” landraces, the two red Spanish commercial varieties, and the two outlier accessions from the “Aglio Rosso di Castelliri” landrace. The accessions belonging to the “Aglio Rosso di Proceno” landrace showed significantly higher values for bulb total phenolics compared to the accessions of the “Aglio Rosso di Castelliri” landrace (Table 7). However, this difference can be attributed only to the higher amounts detected in two of the six accessions (R_PROC_5 and R_PROC_8) from the “Aglio Rosso di Proceno” landrace (Table S16). Conversely, no significant differences were found between the two landraces and the control accessions regarding antioxidant activity (Table 7). On the other hand, accessions from both the “Aglio Rosso di Proceno” and “Aglio Rosso di Castelliri” landraces were characterized by significantly higher levels of total phenolics compared to the group of control accessions (Table 7).

## 4. Discussion

Although most garlic varieties are propagated asexually, considerable morphological variation exists both within and among them, largely attributable to different environmental conditions. This variability poses challenges for their identification and conservation [50,55]. Evaluating the levels of genetic variation within and between garlic varieties is essential for accurate phenotypic identification, the development of core germplasm collections, and for selection and breeding purposes [56,88]. Furthermore, assessing genetic diversity and estimating the degree of relatedness between garlic varieties are essential steps in determining their authenticity and distinctiveness.

In Italy, the significant variability in pedo-climatic contexts, coupled with historical and cultural fragmentation, has led to the development of several garlic landraces over the centuries. These landraces, distinguished by their marked adaptation to the specific environmental conditions of their cultivation areas, have been preserved for their organoleptic attributes, as well as for cultural or sentimental reasons [26,27,28]. Given the strong connection between the nutritional and organoleptic qualities of these products, their geographical origin, and cultivation practices, it is crucial to characterize these agro-foods and develop traceability tools for their authentication. The combination of morphological, chemical, and genetic analyses provides a robust framework for establishing the authenticity of typical garlic products and protecting them [24,26,28,60,89], thus providing the opportunity to stimulate their cultivation and the preservation of local ecosystems [90].

The present study aimed to assess the intra-varietal diversity and distinctiveness of two red garlic landraces (“Aglio Rosso di Castelliri” and “Aglio Rosso di Proceno”) still cultivated in the Lazio region of central Italy, based on SSR and ISSR molecular markers, plant (bulb and clove) traits, and bulb total phenolics and antioxidant properties. Although garlic landraces are considered distinct but variable populations, few studies focusing on the genetic and phenotypic diversity in garlic landraces have addressed intra-landrace diversity [24,56].

First, using SSR and ISSR markers, we aimed to molecularly characterize and assess the genetic diversity and structure of a garlic collection comprising 11 accessions of “Aglio Rosso di Castelliri” and nine accessions of “Aglio Rosso di Proceno” landraces, and 15 garlic accessions used as controls. The controls included eight accessions of red-type garlic landraces cultivated in Italy, four Spanish red garlic commercial varieties, and two accessions of white garlic. In addition, an accession belonging to the species *A. ampeloprasum* var. *holmense* was used as an outgroup genotype.

SSRs are regarded as ideal DNA markers because of their stability and reproducibility [35,56]; consequently, they have been extensively utilized to evaluate genetic diversity in local and worldwide garlic collections [34,35,52,54,55,56,91]. The 13 SSR markers (seven genomic and six EST) used in this study revealed a mean PIC value of 0.38, with a range of 0.11–0.57, which is similar to the mean PIC values reported by Li et al. [39] (0.36, ranging from 0.22 to 0.49), Barboza et al. [34] (0.38, ranging from 0.30 to 0.54), and Papaioannou et al. [56] (0.47, ranging from 0.13 to 0.68), using 29 (22 EST and seven genomic), 10 (EST), and five (genomic) SSR markers in 127, 73, and 27 garlic accessions of Korean, Argentine, and Greek origin, respectively. However, the level of polymorphism detected in this study is lower than the mean PIC of 0.72 (range = 0.65–0.80) reported by Chen et al. [35] using eight genomic SSRs in 39 accessions of different Asian origins, and lower than the mean PIC of 0.63 (range = 0.23–0.84) reported by Zhao et al. [91] with eight genomic SSRs in a world garlic collection of 613 accessions. It is also lower than the mean PIC of 0.60 (range = 0.20–0.86) obtained by Ipek et al. [52] using 26 EST-SSRs in 31 garlic accessions. Variations in the mean PIC values observed across different genetic diversity studies in garlic can be attributed to multiple factors. These include the number and type of SSR markers utilized (e.g., EST or genomic SSRs), their distribution and abundance within the genome, the number and geographic origin of the accessions, and the extent of genetic variation present in the analyzed germplasm collections [34,55,56]. In our collection, 32 of the 35 analyzed accessions were of the red garlic type, and among these, nine and 11 potentially belonged to the same landrace. Presumably, the overrepresentation of genetically similar garlic accessions from the two landraces “Aglio Rosso di Castelliri” and “Aglio Rosso di Proceno” may have contributed to the lower overall genetic diversity found in our collection, as compared to other studies of national or world garlic collections.

Other estimated parameters for the codominant SSR markers, including observed heterozygosity (Ho), expected heterozygosity (He), and the number of alleles per locus (Na), demonstrated comparative levels of genetic variation between our collection and previously characterized garlic germplasm collections [34,54,56,57].

Compared to SSR markers, ISSRs exhibited a lower mean PIC value (0.18), which closely aligns with the mean PIC value (0.19) reported by Kiraç et al. [48] using 10 ISSR primers in 39 Turkish garlic accessions. However, this value is significantly lower than those reported by Rakes et al. [49] (0.65) and Chen et al. [35] (0.72), who used 10 and 17 ISSR primers in 131 and 39 garlic accessions of Indian and Chinese origin, respectively. Nevertheless, in these latter studies, it is challenging to understand how PIC values greater than 0.5 could be obtained for ISSR primers. According to Serrote et al. [74], for dominant markers, the PIC value indicates the probability of finding the marker in two different states (present/absent) in two randomly selected individuals from a population. The PIC value ranges from zero for monomorphic markers to 0.5 for markers present in 50% of individuals and absent in the remaining 50%. Despite the higher number of amplicons detected by the ISSR primers, and consequently the potential to explore a broader region of the garlic genome, these markers were less informative (in terms of PIC and He values) than the codominant SSR markers. This reduced informativeness is attributed to the uncertain allelic phase associated with ISSR primers [64,92].

When assessing the genetic diversity and population structure of a garlic germplasm collection, SSR and ISSR markers have proven to be highly effective in resolving issues related to the nomenclature of accessions and identifying potential redundancies, thus facilitating the construction of core garlic germplasm collections [34,35,55,56]. In this study, molecular analyses employing the two types of molecular markers were essential for accurately assigning accessions to their respective landrace, resolving common challenges in the analysis of local varieties, including genetic variability within apparently uniform materials, inconsistencies between local names and genetic profiles, issues of homonymy, and contamination with foreign genetic material. For instance, two accessions of the landrace “Aglio Rosso di Castelliri” (R_CAST_1 and R_CAST_8), which were assigned to this landrace based on the declarations of local farmers, showed a clear genetic similarity with commercial Spanish red garlic varieties used as controls, indicating a likely case of genetic contamination. This contamination may have arisen from the introduction into cultivation of phenotypically similar but genetically distinct material from most accessions belonging to the “Aglio Rosso di Castelliri” landrace.

Four distinct groups of genetically related accessions were consistently identified by all clustering methods employed (UPGMA, PCoA, and STRUCTURE), indicating convergent results regarding the genetic relatedness of the garlic accessions. The accessions correctly assigned to “Aglio Rosso di Proceno” and “Aglio Rosso di Castelliri” were grouped into two distinct clusters, indicating genetic distinguishability between them and, in general, compared to the red garlic varieties/landraces used as controls. Additionally, our findings allowed for hypotheses regarding the origin of the two landraces. According to the clustering results, the two landraces from the Lazio region may have originated from genotypes of the renowned and widely cultivated landrace “Aglio Rosso di Sulmona”, likely due to the exchange of “seeds” among farmers in central Italy. Alternatively, all three landraces could have originated from a common ancestral population cultivated in central Italy in the past. Although it is not possible to determine with certainty when such exchanges or divergence events may have occurred, this hypothesis is supported by the observed genetic similarities and the traditional agricultural practices historically documented in central Italy. Over time, these materials evolved under the specific pedoclimatic conditions of their cultivation areas, and their genetic and phenotypic differentiation could be the result of natural selection and/or indirect selection exerted by farmers in their respective cultivation environments. According to Pooler and Simon [93] and Casals et al. [24], novel phenotypic variation in garlic can arise from somatic mutations as well as microbial infections. These infections can influence plant fitness and the appearance of bulbs and cloves, and they can be transmitted through asexual propagation across generations. Both somatic mutations and microbial infections, coupled with indirect selection by farmers, may have acted as the driving forces behind the phenotypic and genetic differentiation of the “Aglio Rosso di Proceno” and “Aglio Rosso di Castelliri” landraces from their ancestral material.

Molecular analyses using both SSR and ISSR markers revealed a substantial level of intra-population diversity within the two garlic landraces from the Lazio region. Indeed, AMOVA analysis conducted to partition the molecular markers diversity among and within the two landraces revealed that the majority of the genetic variability determined by SSR markers was ascribed to differences among landraces (78%), but a significant portion of genetic variability (22%) was observed within them. Conversely, for ISSR primers, only 45% of the total variation was among landraces, with the largest portion (55%) attributed to differences within them. Moreover, according to UPGMA and PCoA analyses, the combined use of SSR and ISSR markers allowed the identification of distinct genetic profiles associated with different genotypes for each landrace. These findings indicate that, despite the asexual reproduction of the species, the two landraces consist of a set of genetically distinct clones. Consequently, sampling one or a few individuals from each landrace, as is commonly performed in genetic diversity studies of garlic collections, could provide incomplete information, as it would not adequately represent their entire genetic background. Nevertheless, as previously indicated, clustering analyses demonstrated that all the accessions within each landrace were grouped together, sharing a similar genetic pool. This implies that the observed genetic background within each of the two landraces likely reflects their true variability. The detected intra-varietal genetic variability within the two landraces, on the one hand, provides valuable insights for devising effective in situ/on-farm conservation strategies and, on the other hand, underscores the necessity for clonal selection programs to supply farmers with more uniform and selected material, especially in view of a revival of the cultivation of these landraces. This latter activity could be particularly relevant for the landrace “Aglio Rosso di Proceno”, which exhibited greater genetic diversity compared to “Aglio Rosso di Castelliri”, and for which two distinct groups were identified both genetically and in terms of certain phenotypic traits of the bulbs and cloves.

Several previous studies demonstrated that the variation in bulb and clove morphological traits, as well as their chemical composition, including total phenolic content and antioxidant properties, in addition to genotypic differences, is largely influenced by the environmental conditions of the growing location, including soil properties, cultivation practices, and fertilization regimes [24,94,95,96,97]. In this study, considering that all the red garlic accessions tested were cultivated at the same location and under identical cultivation practices, any observed variation in the morphological and chemical traits of bulbs and cloves can be primarily attributed to differences in the genetic background of the accessions.

The 28 red garlic accessions tested in the same experimental field exhibited relatively high diversity among the investigated biometrical traits of bulbs and cloves and the flesh clove color parameters, whose value ranges were in substantial accordance with those reported in literature for several garlic landraces and commercial varieties [25,28,55,85,97]. The dry matter content of the bulbs of the analyzed accessions was also consistent with the values reported in various landraces of Italian, Greek, and Spanish origin [28,95,98]. Clustering and PCoA analyses divided the 28 accessions into four clusters, with the nine accessions assigned to the “Aglio Rosso di Castelliri” and the nine of “Aglio Rosso di Proceno” placed in distinct clusters, indicating the distinctiveness of the two landraces in the bulb and clove morphological traits. In accordance with the molecular analyses, the two accessions considered atypical for the “Aglio Rosso di Castelliri” landrace (R_CAST_1 and R_CAST_8) grouped together with the two Spanish red garlic commercial varieties, forming a cluster distinct from the one containing the nine representative accessions of “Aglio Rosso di Castelliri”.

Regarding the two landraces cultivated in the Lazio region, the phenotypic uniqueness of “Aglio Rosso di Castelliri”, based on the nine accessions assigned through genetic analysis, seems related to the smaller size of the bulbs and cloves, but with a higher concentration of dry matter, and the appearance of the clove flesh. The color profile of peeled cloves is characterized by greater lightness (L*), higher a* values, and lower b* values compared to the controls and the accessions of “Aglio Rosso di Proceno”. The color of clove flesh in garlic is regarded as an important sensory indicator for consumers, strongly associated with taste, appearance, and flavor. In this context, Liu et al. [87] observed that high a* values of peeled cloves are associated with a softer texture, spicier taste, yellowish appearance, intense garlic flavor, and a higher overall impression. Preliminary results on the sensory profile of the “Aglio Rosso di Castelliri” landrace appear to corroborate its pronounced spiciness and robust flavor, although detailed data are not reported here. On the other hand, in agreement with the genetic analyses, two phenotypic groups can be distinguished within the landrace “Aglio Rosso di Proceno” based on the size of the bulbs and cloves. Four accessions (R_PROC_3 and R_PROC_7–9) exhibited bulb dimensional traits and bulb and clove weights similar to those of the nine accessions assigned to the landrace “Aglio Rosso di Castelliri” but significantly lower than those of the other five accessions (R_PROC_1–2 and R_PROC_4–6) belonging to “Aglio Rosso di Proceno”. Interestingly, all nine accessions of “Aglio Rosso di Castelliri”, as well as four of the nine assigned to the “Aglio Rosso di Proceno” landrace (specifically, R_PROC_3 and R_PROC_7–9), exhibited a high solids content (DM > 43%). These accessions are valuable for both fresh consumptions, due to their anticipated longer shelf life and superior postharvest quality, and for industrial dehydration, a rapidly expanding sector in the processed food market for garlic products [99].

A significant variation was also detected in the bulb concentration of total phenolics and their antioxidant activity among 18 selected red garlic accessions, with a strong positive correlation between these two parameters. Notably, the accessions of the two landraces from the Lazio region exhibited significantly higher phenolic content compared to the group of accessions used as controls. The total phenolic content of the studied accessions, ranging from 33.02 to 76.24 mg GAE/100 g fw with a mean of 42.59 mg GAE/100 g fw, was comparable to the values reported by Avgeri et al. [100] (range = 16.0–81.9 mg GAE/100 g fw; mean = 37.19) and Barboza et al. [34] (range = 13.6–98.7 mg GAE/100 g fw; mean = 36.8), who evaluated 34 Greek garlic landraces/varieties and 73 garlic accessions of Argentine origin, respectively. In contrast, Bushal et al. [21] (range = 26.40–233.70 mg GAE/100 g fw; mean = 100.82) and Hirata et al. [101] (range = 52.59–137.52 mg GAE/100 g fw; mean = 83.58), who evaluated 26 Indian garlic accessions and a large worldwide garlic collection, respectively, reported higher values for total phenolic content in the bulbs than those observed in this study. As discussed previously, variations in the bulb total phenolic content observed across different studies in garlic could be attributed to the different genotypes analyzed and the environmental conditions in which they were grown. The total phenolic content in *Allium* species is likely associated with its potent antioxidant, anti-inflammatory, and anticancer properties [16]. Additionally, the total phenolic concentration in garlic has been strongly correlated with antioxidant capacity, independent of the specific composition of individual phenolic compounds [16,102]. In this study, there is strong evidence of such correlation between the total phenolic content and the antioxidant properties measured by the DPPH assay.

We observed moderate to weak, yet significant, correlations between the genetic groupings of garlic accessions based on SSR and ISSR markers and some of the evaluated morphological and color traits of bulbs and cloves. However, no significant correlations were identified between the genetic clustering of the garlic accessions and their bulb total phenolic content and antioxidant activity. A few authors have reported a moderate correlation between genetic clustering based on molecular markers and the phenotypic characteristics of garlic accessions [55,103], while other studies have found no such correlations [34,54,56].

The lack of a correlation between the total phenolic content and the genetic background of garlic accessions observed in this study is not entirely surprising. Phenolic compounds, which are a class of secondary metabolites, are more significantly affected by external stressors than by genetic factors [104]. Although the genetic makeup of the plant provides the basis for phenolic biosynthesis, their accumulation is strongly modulated by environmental conditions. Therefore, while genetic factors are important, the total phenolic content in garlic is predominantly shaped by environmental influences, making it challenging to establish a direct correlation between phenolic levels and genetic background alone [105]. Moreover, the absence of correlation between phenotypic variation and molecular marker patterns may be attributed to the lack of association between genotyping markers and morphological traits, as well as to potential epigenetic variations within garlic lines that have been cultivated in certain regions for an extended period. Epigenetic variations represent alterations in gene expression patterns that do not involve changes in the DNA sequence. Barboza et al. [34] addressed this issue to explain the observed discrepancies in the levels of genetic and phenotypic variation in the Argentine garlic collection. Drawing on the studies by Gimenez and García-Lampasona [106] and Gimenez et al. [107], they proposed that frequent and dynamic epigenetic modifications occurring in both coding and non-coding regions of the garlic genome, even under standard field cultivation conditions, might account for the discrepancies observed between molecular and phenotypic variation, as opposed to the less frequent occurrence of genetic changes.

## 5. Conclusions

This study provides valuable insights into the genetic and phenotypic diversity of two red garlic landraces from the Lazio region, specifically “Aglio Rosso di Castelliri” and “Aglio Rosso di Proceno”. Through the application of SSR and ISSR molecular markers, significant intra-varietal diversity was detected, revealing the presence of genetically distinct clones within each landrace, despite garlic’s characteristic asexual reproduction. The use of SSR and ISSR markers was also instrumental in distinguishing these landraces, identifying possible instances of genetic contamination, and providing clues to their potential historical origins. Phenotypic analyses further confirmed the uniqueness of these landraces, particularly with regard to bulb and clove morphology, dry matter content, and chemical composition, including total phenolics and antioxidant activity. The findings underscore the importance of comprehensive characterization for accurate landrace identification and conservation. The observed genetic and phenotypic diversity between the two examined landraces can be attributed to their long-term adaptation to specific environmental conditions (pedo-climatic factors and less intensive agronomic practices), as well as the likely indirect selection performed by farmers over generations.

Beyond their genetic and phenotypic distinctiveness, these garlic landraces hold significant agronomic and economic potential due to their adaptation to local conditions, suggesting greater resilience to environmental stressors and potential advantages over modern cultivars in specific growing environments. Additionally, their unique morphological and biochemical traits may contribute to distinctive sensory and nutritional properties, making them highly valuable for culinary applications. Traditional landraces are often prized for their superior organoleptic qualities, and these local varieties could serve a niche market for high-value, region-specific products. This could further incentivize their cultivation, enhancing both their economic viability and their role in preserving local agricultural biodiversity. Despite their importance, these traditional garlic landraces face a high risk of genetic erosion due to the increasing dominance of commercial varieties and the shift toward intensive farming systems. Therefore, targeted conservation efforts are essential, including both in situ and ex situ strategies.

Finally, the study provides a valuable framework for future breeding programs aimed at improving garlic landraces while preserving their unique genetic traits. Future research should explore their agronomic performance under different cultivation conditions, their potential disease resistance, and their yield characteristics compared to modern varieties. Moreover, consumer awareness and market strategies promoting the use of these traditional varieties in specialized culinary applications could further enhance their commercial value.

In conclusion, the comprehensive characterization of these two garlic landraces not only contributes to their conservation but also highlights their potential for sustainable agricultural practices, gastronomic innovation, and cultural heritage preservation. Strengthening the link between traditional agricultural practices, scientific research, and market opportunities is fundamental to preserving and enhancing these valuable genetic resources for the future.

## Figures and Tables

**Table 1:** Genetic diversity parameters from the 13 SSR loci and the 10 ISSR primers used for the analysis of the 34 garlic accessions and the landrace “AGLIONE” of *A. ampelosarum* var. *holmense*. Number of alleles per locus (N_A_); major allele frequency (MAF); expected heterozygosity (He); observed heterozygosity (Ho); polymorphism information content (PIC); total bands (TB); polymorphic bands (PB); polymorphism percentage (% Pol). The values calculated for the 34 garlic accessions are provided in parentheses.

SSR	No. Allele			MAF	He	PIC	Ho
GB_ASM_040	5 (5)			0.66 (0.68)	0.65 (0.63)	0.51 (0.48)	0.91 (0.94)
GB_ASM_078	6 (4)			0.80 (0.82)	0.59 (0.58)	0.33 (0.29)	1.00 (1.00)
GB_ASM_059	4 (3)			0.86 (0.85)	0.60 (0.60)	0.24 (0.24)	1.00 (1.00)
Asa_24	5 (3)			0.46 (0.47)	0.67 (0.65)	0.49 (0.45)	0.97 (1.00)
Asa_17	4 (4)			0.89 (0.91)	0.19 (0.14)	0.21 (0.16)	0.03 (0.03)
Asa_25	5 (4)			0.89 (0.91)	0.19 (0.14)	0.21 (0.16)	0.06 (0.06)
Asa_10	5 (5)			0.71 (0.74)	0.38 (0.35)	0.45 (0.41)	0.11 (0.12)
AS_5944	7 (5)			0.46 (0.47)	0.56 (0.54)	0.60 (0.57)	0.20 (0.18)
AS_739	5 (5)			0.54 (0.56)	0.73 (0.74)	0.54 (0.51)	0.94 (0.97)
AS_589	4 (4)			0.57 (0.59)	0.53 (0.50)	0.47 (0.44)	0.03 (0.03)
AS_11065	6 (6)			0.57 (0.59)	0.65 (0.63)	0.57 (0.54)	0.86 (0.88)
AS_987	7 (6)			0.49 (0.50)	0.68 (0.67)	0.58 (0.55)	0.97 (0.97)
AS_30	5 (3)			0.91 (0.94)	0.14 (0.09)	0.16 (0.11)	0.06 (0.06)
MEAN	5.23 (4.38)			0.68 (0.69)	0.50 (0.48)	0.41 (0.38)	0.55 (0.56)
ST. DEV.				0.17 (0.18)	0.21 (0.23)	0.16 (0.16)	0.45 (0.46)
ISSR	TB	PB	% Pol	MAF	He	PIC	
UBC_832	20 (17)	20 (17)	100% (100%)	0.86 (0.86)	0.20 (0.21)	0.20 (0.21)	
UBC_834	16 (13)	16 (13)	100% (100%)	0.93 (0.93)	0.14 (0.13)	0.14 (0.13)	
UBC_842	17 (13)	17 (12)	100% (92%)	0.90 (0.88)	0.16 (0.18)	0.16 (0.18)	
UBC_840	17 (14)	17 (13)	100% (93%)	0.86 (0.87)	0.19 (0.20)	0.19 (0.20)	
UBC_850	17 (14)	17 (10)	100% (71%)	0.90 (0.89)	0.15 (0.15)	0.15 (0.15)	
UBC_851	22 (20)	22 (20)	100% (100%)	0.84 (0.83)	0.23 (0.23)	0.23 (0.23)	
UBC_857	16 (10)	16 (9)	100% (90%)	0.92 (0.91)	0.12 (0.16)	0.12 (0.16)	
UBC_881	22 (17)	22 (14)	100% (82%)	0.95 (0.94)	0.08 (0.09)	0.08 (0.09)	
UBC_848	20 (17)	20 (15)	100% (88%)	0.77 (0.75)	0.30 (0.33)	0.30 (0.33)	
UBC_860	14 (11)	14 (8)	100% (73%)	0.94 (0.94)	0.10 (0.09)	0.10 (0.09)	
TOT	181 (146)	181 (131)					
MEAN	18.1 (14.6)	18.1 (13.1)	100% (89%)	0.89 (0.88)	0.17 (0.18)	0.17 (0.18)	
ST. DEV.	2.73 (3.10)			0.06 (0.06)	0.06 (0.08)	0.06 (0.08)	

**Table 2:** Genetic diversity parameters of SSR and ISSR markers of the two landraces “Aglio Rosso di Castelliri” and “Aglio Rosso di Proceno”. N: number of accessions per landrace; Ne: number of effective alleles; Npa: number of private alleles; I: Shannon index; He: expected heterozygosity; Ho: observed heterozygosity. The asterisk (*) indicates significant differences at *p ≤* 0.01.

SSR Markers						
Pop	N	Ne	Npa	I	He	Ho
Castelliri	9	1.684	4.000	0.485	0.311	0.548
Proceno	9	1.720	5.000	0.515	0.326	0.556
*p*-value Kruskal–Wallis Test		>0.01		>0.01	>0.01	>0.01
ISSR Markers						
Pop	N	Ne	Npa	I	He	
Castelliri	9	1.023	0.000	0.026	0.016	
Proceno	9	1.199	9.000	0.150	0.105	
*p*-value Kruskal–Wallis Test		<0.01 *		<0.01 *	<0.01 *	

**Table 3:** Analysis of molecular variance for the partitioning of SSR and ISSR markers diversity of the two landraces “Aglio Rosso di Castelliri” and “Aglio Rosso di Proceno”. *p* (*Φ*)-*Φ*-statistical probability level after 9999 permutations.

SSR Markers	df	SS	MS	Est. Var.	%	Fst	*Φ*-Statistic	*p* (*Φ*)
Among pops	1	43.389	43.389	4.674	78%	0.227	0.780	<0.001
Within pops	16	21.111	1.319	1.319	22%			
Total	17	64.500		5.994	100%			
ISSR Markers	df	SS	MS	Est. Var.	%	Fst	*Φ*-Statistic	*p* (*Φ*)
Among pops	1	28.500	28.500	2.792	45%	0.345	0.453	<0.001
Within pops	16	54.000	3.375	3.375	55%			
Total	17	82.500		6.167	100%			

**Table 4:** The correlation between genetic and phenotypic (morphological and color traits) distance matrices using the Mantel test. Refer to Table S5 for acronyms of morphological traits. ^a^ Data in the phenotypic matrix were determined by considering all the 17 morphological and color traits of bulbs and cloves. ^b^ r(xy) correlation value between the genetic (x) and phenotypic (y) matrices. ^c^ *p*-value calculated using the distribution of r (xy) estimated from 9999 permutations. *p* < 0.05 was considered significant.

	GD vs. Phenotypic ^a^	GD vs. W	GD vs. De	GD vs. Dp	GD vs. T	GD vs. NCLBulb	GD vs. CLW	GD vs. DM	GD vs. L*	GD vs. a*	GD vs. b*
r(xy) ^b^	0.512	0.45	0.44	0.40	0.42	0.40	0.18	0.38	0.29	0.29	0.18
*p*-value ^c^	0.00	0.00	0.00	0.00	0.00	0.00	0.08	0.00	0.00	0.00	0.08

**Table 5:** ANOVA of the main morphological and color traits of bulbs and cloves in the four clusters identified by cluster and PCoA analyses. Refer to Table S5 for acronyms of morphological traits. The values are means ± standard deviations for each trait considered. Different letters indicate significant differences between clusters at *p* < 0.05.

	W	De	Dp	T	NCLBulb	CLW	DM	L*	a*	b*
CLI	23.60 ± 0.41 ^b^	40.30 ± 1.53 ^b^	32.55 ± 1.24 ^b^	36.00 ± 1.00 ^b^	10.86 ± 0.45 ^b^	2.18 ± 0.11 ^a^	41.15 ± 2.23 ^bc^	72.05 ± 0.64 ^b^	−1.75 ± 0.16 ^b^	24.29 ± 2.15 ^a^
CLII	26.16 ± 0.55 ^a^	42.27 ± 0.96 ^a^	33.98 ± 0.78 ^a^	38.02 ± 1.39 ^a^	12.15 ± 0.42 ^a^	2.15 ± 0.13 ^a^	39.96 ± 2.77 ^c^	70.13 ± 2.48 ^c^	−1.07 ± 0.21 ^a^	21.95 ± 0.90 ^b^
CLIII	17.01 ± 0.62 ^c^	36.10 ± 0.47 ^c^	29.57 ± 1.05 ^c^	31.06 ± 0.34 ^c^	9.50 ± 0.29 ^c^	1.79 ± 0.05 ^b^	43.95 ± 0.90 ^ab^	72.85 ± 0.06 ^b^	−1.69 ± 0.15 ^b^	25.34 ± 1.09 ^a^
CLIV	17.69 ± 0.40 ^c^	36.10 ± 0.47 ^c^	30.06 ± 0.51 ^c^	32.16 ± 0.74 ^c^	10.37 ± 0.76 ^bc^	1.72 ± 0.11 ^b^	44.60 ± 1.10 ^a^	77.76 ± 0.94 ^a^	−1.20 ± 0.09 ^a^	22.28 ± 0.70 ^b^

**Table 6:** Differences in total phenolics and antioxidant activity of bulbs (means of eight measurements) among the 18 garlic accessions. Mean values and standard deviations in columns with different letters are significantly different at *p* < 0.05 according to Tukey’s HSD test; *** indicates significant difference at *p* < 0.001 following ANOVA analysis; CV = coefficient of variation expressed as a percentage.

Accession	Total Phenolics mg GAE/100 g fw	Antioxidant Activity mM TE/g fw
R_PROC_3	38.69 ± 1.21 ^e,f^	3.05 ± 0.26 ^e–g^
R_PROC_5	76.24 ± 4.38 ^a^	7.81 ± 0.28 ^a^
R_PROC_6	45.75 ± 1.23 ^c,d^	5.87 ± 0.51 ^c,d^
R_PROC_7	38.61 ± 1.59 ^e,f^	3.47 ± 0.23 ^e–h^
R_PROC_8	56.18 ± 1.36 ^b^	6.09 ± 0.41 ^b^
R_PROC_9	38.14 ± 1.96 ^e,f^	4.85 ± 0.50 ^e,f^
R_CAST_1	33.02 ± 1.56 ^f^	1.96 ± 0.23 ^h^
R_CAST_3	37.99 ± 1.40 ^e,f^	4.69 ± 0.38 ^e,f^
R_CAST_4	37.82 ± 1.38 ^e,f^	3.77 ± 0.34 ^d,e^
R_CAST_5	48.46 ± 2.23 ^c^	6.08 ± 0.29 ^b,c^
R_CAST_6	48.92 ± 3.57 ^c^	4.59 ± 0.27 ^d^
R_CAST_8	33.09 ± 2.01 ^f^	2.33 ± 0.40 ^g,h^
R_CAST_9	40.21 ± 1.82 ^d,e^	4.27 ± 0.26 ^d,e^
R_SULM_4	40.61 ± 1.82 ^d,e^	3.85 ± 0.64 ^f,g^
R_SULM_3	37.79 ± 2.36 ^e,f^	3.61 ± 0.44 ^e,f^
R_SORA_1	40.69 ± 1.06 ^d,e^	2.9 ± 0.41 ^f–h^
R_SPA_2	38.41 ± 1.49 ^e,f^	3.63 ± 0.58 ^f–h^
R_SPA_4	35.52 ± 2.13 ^e,f^	3.62 ± 0.35 ^f–h^
CV	24.07	39.67
*p*	***	***

**Table 7:** ANOVA of the content of total phenolics and antioxidant activity of bulbs across three groups of garlic accessions: landrace accessions of “Aglio Rosso di Proceno”, “Aglio Rosso di Castelliri”, and red-type garlic accessions used as controls. AO: antioxidant activity; TPC: total phenolic content. The values are means ± standard deviations for each trait considered. Different letters indicate significant differences between clusters at *p* < 0.05. ns means no statistical differences.

	AO (TE/g fw)	TPC (GAE/100 g fw)
“Aglio Rosso di Proceno”	5.19 ± 1.77 ^ns^	48.93 ± 1.96 ^a^
“Aglio Rosso di Castelliri”	3.95 ± 1.47 ^ns^	42.76 ± 2.32 ^b^
Control red-type garlic	3.52 ± 0.36 ^ns^	37.02 ± 2.12 ^c^

## Data Availability

The data contained within the present article and in its Supplementary Materials are freely available upon request to the corresponding authors.

## Supplementary Materials

The following supporting information can be downloaded at https://www.mdpi.com/article/10.3390/plants14081189/s1, Figure S1. Bulbs and cloves from two accessions of the “Aglio Rosso di Castelliri” (R_CAST_2 and R_CAST_11) and “Aglio Rosso di Proceno” (R_PROC_2 and R_PROC_5) landraces, as well as from one accession of the “Aglio Rosso di Sulmona” landrace (R_SULM_2) used as a control. Below are the unpeeled and peeled cloves of two accessions (R_CAST_9 and R_PROC_8) from the “Aglio Rosso di Castelliri” and “Aglio Rosso di Proceno” landraces; Figure S2. Cross-sectional view of bulbs from five accessions of the “Aglio Rosso di Castelliri” (top) and “Aglio Rosso di Proceno” (bottom) landraces; Figure S3. Agarose gel electrophoresis of PCR products by four ISSR primers from representative accessions of “Aglio Rosso di Proceno” and “Aglio Rosso di Castelliri” landraces, along with garlic landraces/varieties used as references. (A) UBC_842, (B) UBC_840, (C) UBC_851, and (D) UBC_857 primers; Figure S4. Genetic clustering of 34 *A. sativum* accessions and the outgroup species *A. ampeloprasum* var. *holmense* (AGLIONE) based on principal coordinate analysis (PCoA) using data from 13 SSR and 10 ISSR markers. The Y and X axes represent the two main coordinate axes, and the percentage values indicate the proportion of the variation in sample genetic composition explained by these axes. The closer the two sample points are, the more similar their genetic composition; Figure S5. Estimation of the optimal number of clusters for the 34 *A. sativum* accessions according to the Evanno method (2005). The graphs on the left display the DeltaK [mean(|L″(K)|)/sd(L(K))] for each value of K. (A) Statistics and results of the Evanno test for the first STRUCTURE analysis. (B, C) Statistics and results of the Evanno test for the second STRUCTURE analysis for the accessions included in each of the two clusters (nine and 24, respectively) identified in the first analysis (A). Table S1. Accession codes, common name of the landrace/commercial variety, and bulbs provenances relative to the 35 garlic accessions analyzed in this study; Table S2. Characteristics of the 13 SSR loci used in this study; Table S3. Characteristics of the 10 ISSR primers used in this study; Table S4. Characteristics of Alvito soil. Table S5. List of the qualitative and quantitative traits recorded for the 28 garlic accessions used in the experimental trial, with their corresponding scales of measurement and acronyms; Table S6. SSR private alleles detected in the 35 garlic genotypes in homozygous (Hom) or heterozygous (Het) status; Table S7. ISSR private bands (alleles) detected in the 35 garlic accessions; Table S8. Posterior membership coefficients (Q) following a STRUCTURE analysis of the 34 *A. sativum* accessions with K = 2 and K = 4; Table S9. SSR private alleles detected in “Aglio Rosso di Castelliri” and “Aglio Rosso di Proceno” landraces; Table S10. ISSR private alleles and their frequencies detected in the “Aglio Rosso di Proceno” landrace. No private alleles were found in the “Aglio Rosso di Castelliri” landrace. Table S11. Morphological qualitative traits of bulbs and cloves retrieved by using UPOV and IPGRI descriptors for the 28 garlic accessions. Extension of the acronyms for the qualitative traits are as indicated in Table S5; Table S12. Differences in 12 morphological quantitative traits of bulbs and cloves (means of four measurements) for the 28 garlic accessions. Refer to Table S5 for acronyms of quantitative traits. Mean values and standard deviations in columns with different letters are significantly different at *p* < 0.05 according to Tukey’s HSD test; *** indicates significant difference at *p* < 0.001 following ANOVA analysis; ns = not significant; CV = coefficient of variation expressed as a percentage; Table S13. Pearson correlation coefficients of morphological quantitative traits of bulbs and cloves. Refer to Table S5 for acronyms of quantitative traits. ** Correlation significant at *p* < 0.01. * Correlation significant at *p* < 0.05; Table S14. Differences in color parameters of peeled cloves among the 28 garlic accessions expressed as average value (from 16 measurements) of the different components according to the CIELab scale. Mean values and standard deviations in columns with different letters are significantly different at *p* < 0.05 according to Tukey’s HSD test; *** indicates significant difference at *p* < 0.001 following ANOVA analysis; CV = coefficient of variation expressed as a percentage; Table S15. Total eigenvalues as well as relative and cumulative proportion of total variance explained by each component and link of the first two PCs with the morphological and color traits of bulbs and cloves. Refer to Table S5 for acronyms of morphological traits; Table S16. The correlation between genetic and phenotypic (antioxidant activity and total phenolics) distance matrices using the Mantel test. AO: antioxidant activity. TPC: total phenolic content. ^a^ r (xy) correlation value between the genetic (x) and phenotypic (y) matrices. ^b^ *p*-value calculated using the distribution of r (xy) estimated from 9999 permutations. *p* < 0.05 was considered significant.
