# Supplementary material for: Genetic and Phenotypic Diversity and Evaluation of Total Phenolics and Antioxidant Properties of Garlic Landraces from Lazio Region (Central Italy): “Aglio Rosso di Proceno” and “Aglio Rosso di Castelliri”
Source: Plants (Basel). 2025 Apr 11;14(8):1189. doi: 10.3390/plants14081189 (PMC12030451; doi:10.3390/plants14081189)
Supplement: Supplementary file 1 [file plants-14-01189-s001.zip › plants-3548789-supplementary.pdf]

Supplementary
Figures and Tables

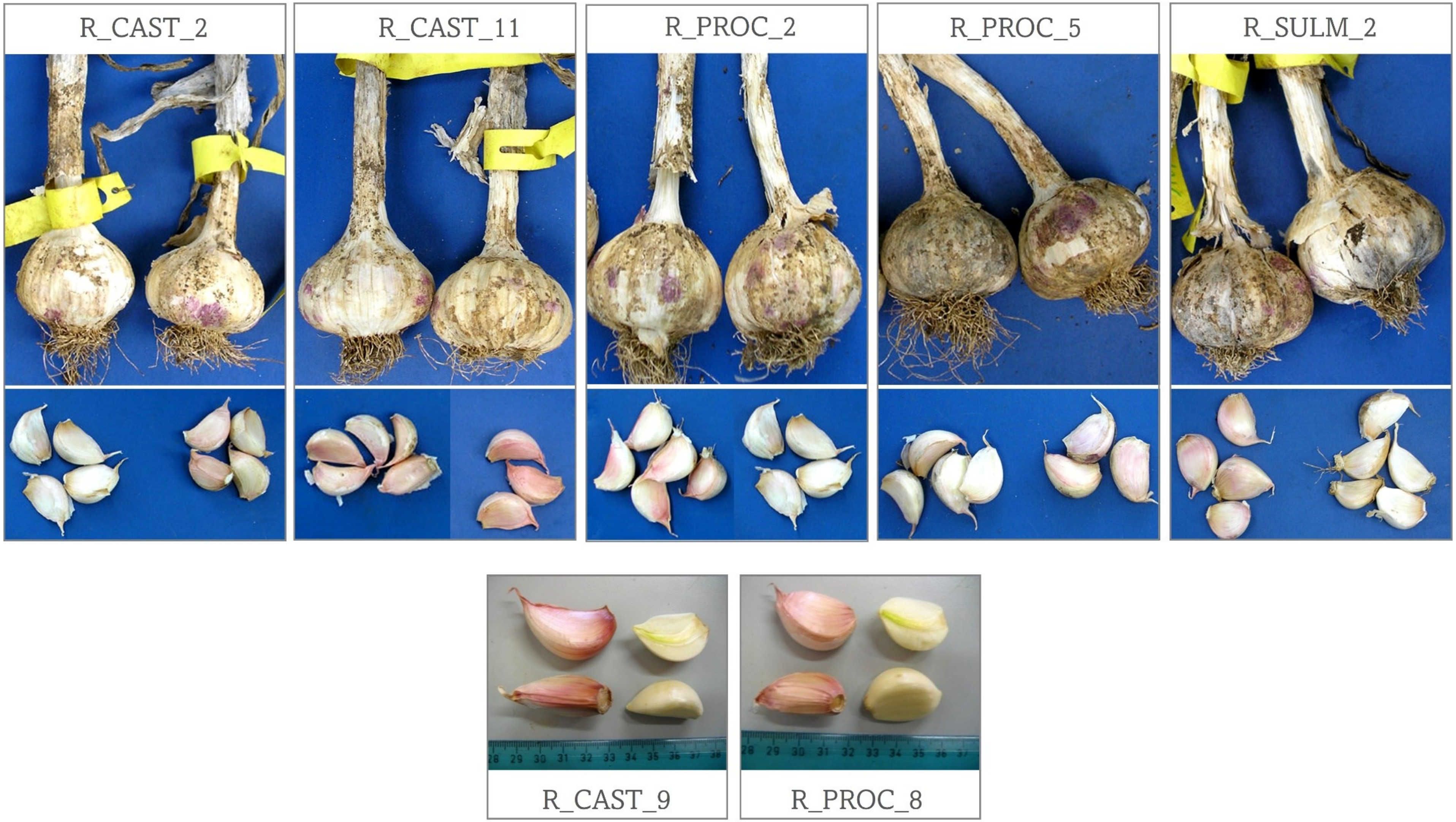

Figure S1. Bulbs and cloves from two accessions of the "Aglio Rosso di Castelliri" (R_CAST_2 and R_CAST_11) and "Aglio Rosso di Proceno" (R_PROC_2 and R_PROC_5) landraces, as well as from one accession of the "Aglio Rosso di Sulmona" landrace (R_SULM_2) used as a control. Below are the unpeeled and peeled cloves of two accessions (R_CAST_9 and R_PROC_8) from the "Aglio Rosso di Castelliri" and "Aglio Rosso di Proceno" landraces.

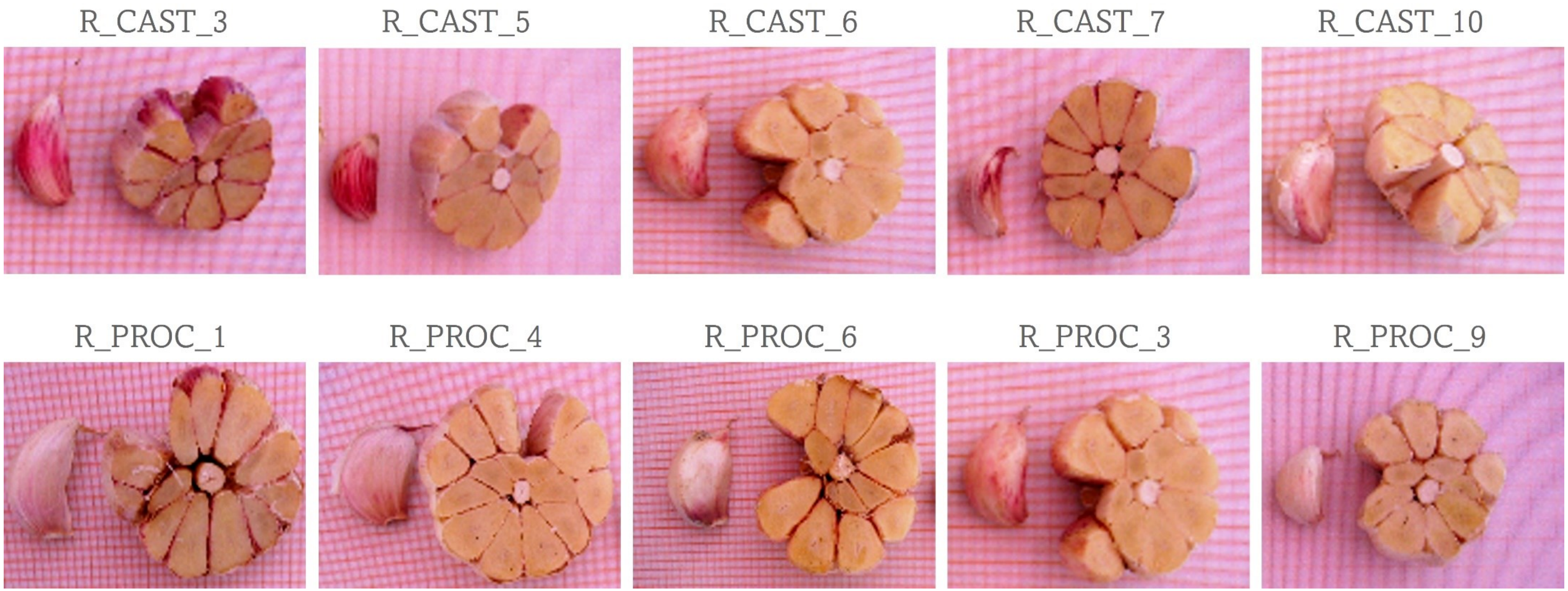

Figure S2. Cross-sectional view of bulbs from five accessions of the "Aglio Rosso di Castelliri" (top) and "Aglio Rosso di Proceno" (bottom) landraces.

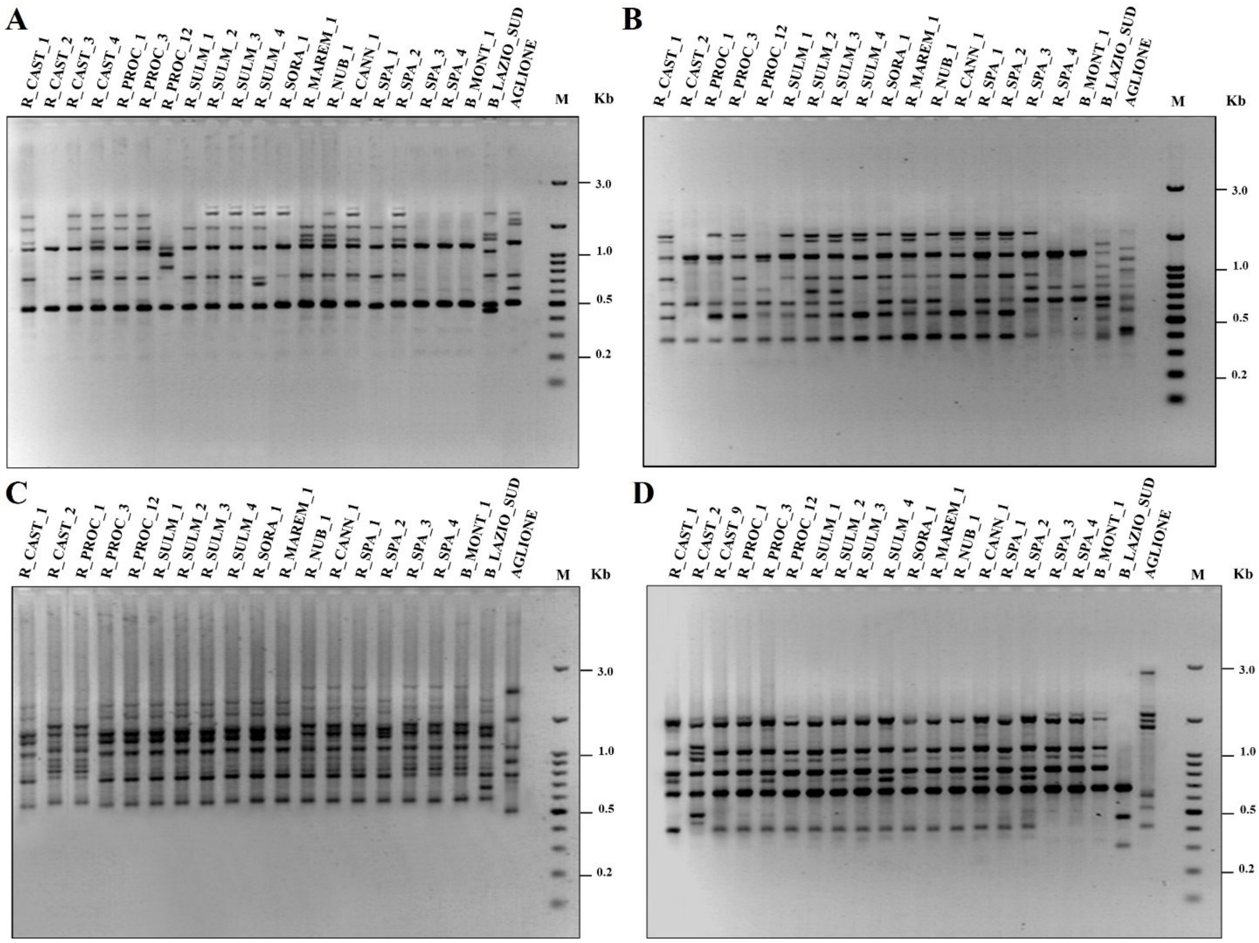

Figure S3. Agarose gel electrophoresis of PCR products by four ISSR primers from representative accessions of "Aglio Rosso di Proceno" and "Aglio Rosso di Castelliri" landraces, along with garlic landraces/varieties used as references. (A) UBC_842, (B) UBC_840, (C) UBC_851, and (D) UBC_857 primers.

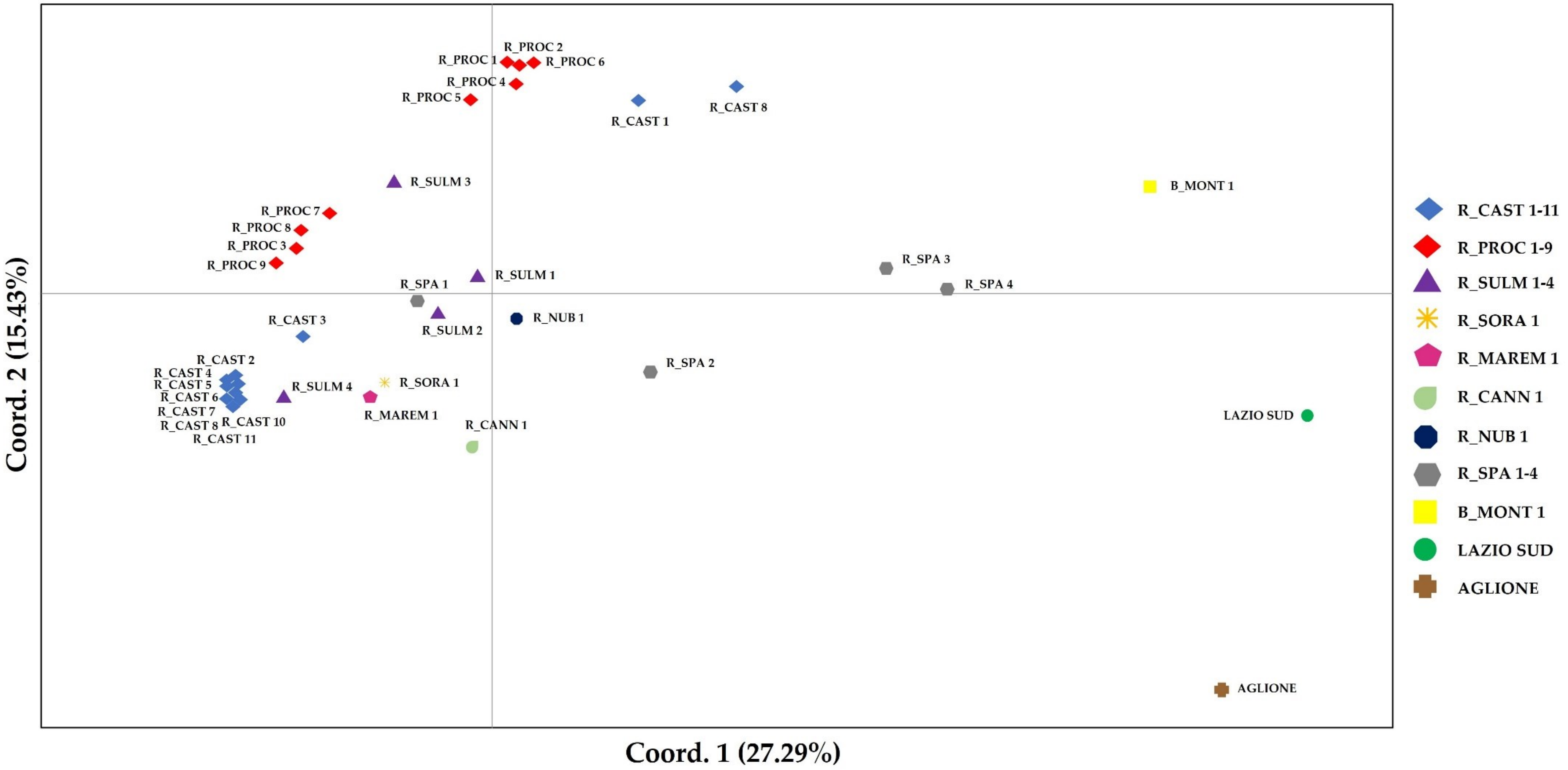

Figure S4. Genetic clustering of 34 *A. sativum* accessions and the outgroup species *A. ampeloprasum* var. *holmense* (AGLIONE) based on principal coordinate analysis (PCoA) using data from 13 SSR and 10 ISSR markers. The Y and X axes represent the two main coordinate axes, and the percentage values indicate the proportion of the variation in sample genetic composition explained by these axes. The closer the two sample points are, the more similar their genetic composition.

A

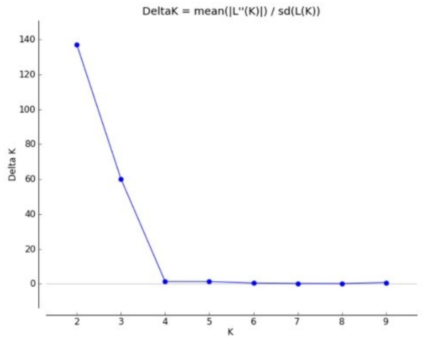

K	Reps	Mean LnP(K)	Stdev LnP(K)	Ln'(K)	|Ln''(K)|	Delta K
1	10	-2555.230000	0.802842	—	—	—
2	10	-1950.260000	2.802856	604.970000	384.110000	137.042375
3	10	-1729.400000	52.083459	220.860000	3129.090000	60.078383
4	10	-4637.630000	3994.181968	-2908.230000	5169.660000	1.294298
5	10	-2376.200000	1794.811046	2261.430000	2289.620000	1.275689
6	10	-2404.390000	555.269105	-28.190000	228.830000	0.412106
7	10	-2661.410000	1041.619545	-257.020000	186.630000	0.179173
8	10	-2731.800000	878.928571	-70.390000	94.560000	0.107586
9	10	-2896.750000	986.521647	-164.950000	687.260000	0.696650
10	10	-2374.440000	447.322002	522.310000	—	—

B

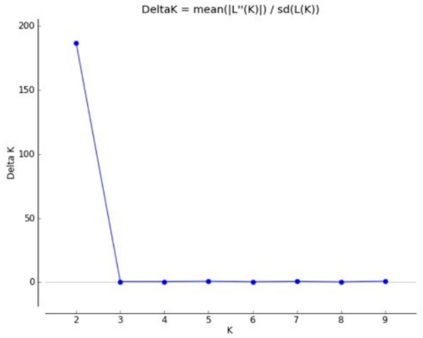

K	Reps	Mean LnP(K)	Stdev LnP(K)	Ln'(K)	|Ln''(K)|	Delta K
1	10	-1424.540000	1.675444	—	—	—
2	10	-1205.250000	1.561516	219.290000	291.780000	186.856832
3	10	-1277.740000	226.003659	-72.490000	92.410000	0.408887
4	10	-1442.640000	533.968728	-164.900000	223.160000	0.417927
5	10	-1384.380000	276.689842	58.260000	190.170000	0.687304
6	10	-1516.290000	309.066100	-131.910000	91.110000	0.294791
7	10	-1557.090000	244.008089	-40.800000	127.530000	0.522647
8	10	-1725.420000	539.069585	-168.330000	98.030000	0.181850
9	10	-1795.720000	522.651322	-70.300000	388.500000	0.743325
10	10	-1477.520000	125.299923	318.200000	—	—

C

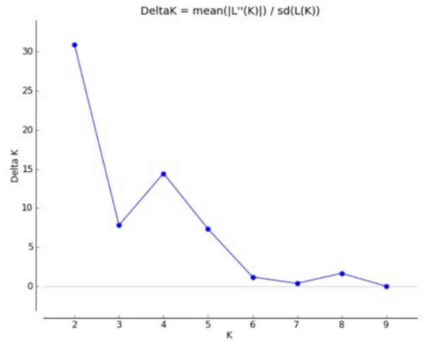

K	Reps	Mean LnP(K)	Stdev LnP(K)	Ln'(K)	|Ln''(K)|	Delta K
1	10	-933.070000	0.666750	—	—	—
2	10	-732.540000	2.855482	200.530000	88.210000	30.891457
3	10	-620.220000	3.014336	112.320000	23.540000	7.809348
4	10	-531.440000	6.183886	88.780000	89.280000	14.437524
5	10	-531.940000	3.172871	-0.500000	23.290000	7.340355
6	10	-555.730000	14.992298	-23.790000	17.900000	1.193946
7	10	-561.620000	9.528763	-5.890000	3.550000	0.372556
8	10	-571.060000	6.537278	-9.440000	10.900000	1.667361
9	10	-569.600000	21.244659	1.460000	0.260000	0.012238
10	10	-568.400000	14.535245	1.200000	—	—

Figure S5. Estimation of the optimal number of clusters for the 34 A. sativum accessions according to the Evanno method (2005). The graphs on the left display the DeltaK [mean(|L''(K)|) / sd(L(K))] for each value of K. (A) Statistics and results of the Evanno test for the first STRUCTURE analysis. (B, C) Statistics and results of the Evanno test for the second STRUCTURE analysis for the accessions included in each of the two clusters (nine and 24, respectively) identified in the first analysis (A).

Table S1. Accession codes, common name of the landrace/commercial variety and bulbs provenances relative to the 35 garlic accessions analyzed in this study.

No.	ARSIAL code	Type of variety	Name	Accession code	Bulbs Provenance
1	VE_325	Landrace	Rosso di Proceno	R_PROC_1	Farmer from Proceno (VT)
2	VE_326	Landrace	Rosso di Proceno	R_PROC_2	Farmer from Proceno (VT)
3	VE_438	Landrace	Rosso di Proceno	R_PROC_3	Farmer from Proceno (VT)
4	VE_503	Landrace	Rosso di Proceno	R_PROC_4	Farmer from Proceno (VT)
5	VE_329	Landrace	Rosso di Proceno	R_PROC_5	Farmer from Proceno (VT)
6	VE_328	Landrace	Rosso di Proceno	R_PROC_6	Farmer from Proceno (VT)
7	VE_327	Landrace	Rosso di Proceno	R_PROC_7	Farmer from Proceno (VT)
8	VE_330	Landrace	Rosso di Proceno	R_PROC_8	Farmer from Proceno (VT)
9	VE_333	Landrace	Rosso di Proceno	R_PROC_9	Farmer from Proceno (VT)
10	VE_310	Landrace	Rosso di Castelliri	R_CAST_1	Farmer from Castelliri (FR)
11	VE_313	Landrace	Rosso di Castelliri	R_CAST_2	Farmer from Castelliri (FR)
12	VE_316	Landrace	Rosso di Castelliri	R_CAST_3	Farmer from Castelliri (FR)
13	VE_492	Landrace	Rosso di Castelliri	R_CAST_4	Farmer from Castelliri (FR)
14	VE_493	Landrace	Rosso di Castelliri	R_CAST_5	Farmer from Castelliri (FR)
15	VE_496	Landrace	Rosso di Castelliri	R_CAST_6	Farmer from Castelliri (FR)
16	VE_498	Landrace	Rosso di Castelliri	R_CAST_7	Farmer from Castelliri (FR)
17	VE_314	Landrace	Rosso di Castelliri	R_CAST_8	Farmer from Castelliri (FR)
18	VE_315	Landrace	Rosso di Castelliri	R_CAST_9	Farmer from Castelliri (FR)
19	VE_497	Landrace	Rosso di Castelliri	R_CAST_10	Farmer from Castelliri (FR)
20	VE_494	Landrace	Rosso di Castelliri	R_CAST_11	Farmer from Castelliri (FR)
21	VE_501	Landrace	Rosso di Sulmona	R_SULM_1	Market in Sora (FR)
22	VE_499	Landrace	Rosso di Sulmona	R_SULM_2	Market in Sora (FR)
23	VE_508	Landrace	Rosso di Sulmona	R_SULM_3	Consortium of red garlic of Sulmona (AQ)
24	VE_507	Landrace	Rosso di Sulmona	R_SULM_4	Farmer from Sulmona (AQ)
25	VE_505	Landrace	Rosso di Cannara	R_CANN_1	Market in Perugia (PG)
26	VE_510	Landrace	Rosso di Nubia	R_NUB_1	Consortium of red garlic of Nubia
27	VE_506	Landrace	Rosso Maremmano	R_MAREM_1	Farmer from Orbetello (GR)
28	VE_509	Commercial variety	Spanish red garlic	R_SPA_1	Market in Sulmona (AQ)
29	VE_500	Commercial variety	Spanish red garlic	R_SPA_2	Market in Sora (FR)
30		Commercial variety	Spanish red garlic CV Morado	R_SPA_3	Commercial reseller
31		Commercial variety	Spanish red garlic CV Gardos	R_SPA_4	Commercial reseller
32	VE_502	Landrace	Rosso di Sora	R_SORA_1	Farmer from Sora (FR)
33		Landrace	Bianco di Montalto	B_MONT_1	Farmer from Montalto di Castro (VT)
34		Commercial variety	Spanish white garlic	B_LAZIO_SUD	Commercial reseller
35		Landrace	Aglione della Chiana	AGLIONE	Farmer from Siena (SI)

Table S2. Characteristics of the 13 SSR loci used in this study.

SSR code	Repeat motif	Forward and Reverse primers	Genebank No.*/GarlicEST db ID**	Ta (°C)	References
GB_ASM_040	$(AC)_6$, $(AC)_{14}$-$(AT)_5$	F: CACAGCAACATGCACCAT<br>R: TGCCGGAACTCGATATT	EU909133*	60	Ma et al. 2009 [33]
GB_ASM_078	$(GT)_{12}$	F: TGTTCCAACCAGATTTAATGC<br>R: AAGTGGCGGTTGTGTCTG	EU909137*	60	Ma et al. 2009 [33]
GB_ASM_059	$(TG)_{11}$, $(TG)_5$	F: CTTGCCGGAACTCGATATT<br>R: CACAGCAACATGCACCAT	EU909135*	61	Ma et al. 2009 [33]
Asa_24	$(GT)_4(GT)_3(GT)_5$	F: TTGTTGTGCCGAGTTCCATA<br>R: CAGCAATTTACCAAAGCCAAG	JN084096*	48	Cunha et al. 2012 [34]
Asa_17	$(CA)_{12}(CT)_{28}$	F: TCCACGACACACACACACAC<br>R: ATGCAGAGAATTTGGCATCC	JN084092*	56	Cunha et al. 2012 [34]
Asa_25	$(CT)_3(CT)_{27}$	F: GCACTTCACTTTCCCCATTC<br>R: GGCGACGGTGAAGAGAGAG	JN084097*	51	Cunha et al. 2012 [34]
Asa_10	$(AC)_7$	F: TTGTTGTTCTGCCATTTT<br>R: GATCTAAGCCGAGAGAAA	JN084089*	48	Cunha et al. 2012 [34]
AS_5944	$(AC)_{28}$	F: AGAGGGTTTTTCGATCTGGA<br>R: AGTGGCATCAAAGCAAGATG	EPP004LLAA12S005944_656**	57	Ipek et al. 2015 [35]
AS_739	$(AGC)_{10}$	F: AACAGGGATCTTTGCTTCAGC<br>R: GATCTGTTGTGGTTGGATGTTC	EPP004LLAA12C000739-1115**	59	Ipek et al. 2015 [35]
AS_589	$(AC)_{10}$	F: TCTTTGCATCTCTGTCTTGCAT<br>R: GAAGGCACGATTACATTTCTCG	EPP005LLAA12C000589-706**	55	Ipek et al. 2015 [35]
AS_11065	$(GA)_{12}$	F: AACAGTCGAAAGCGTGGATTG<br>R: TACGGCTTGCTACCAAAGAC	EPP005LLAA12S011065-751**	57	Ipek et al. 2015 [35]
AS_987	$(AAT)_6$	F: GTACCAACTCTTTCCTAACGC<br>R: TCCAATAGTTGTGATGACAGG	EPP004LLAA12C000987-690**	57	Ipek et al. 2015 [35]
AS_30	$(GCT)_6$-$(AGCAGG)_4$	F: GTGCCTCCTCGACCTTAG<br>R: TAGAAGAACCTGCTGTGACG	EPP005LLAA12C000030-946**	59	Ipek et al. 2015 [35]

Table S3. Characteristics of the 10 ISSR primers used in this study.

ISSR code	Sequence 5'-3'	Ta (°C)	References
UBC_832	$(AG)_8YG$	55	UBC primer dataset
UBC_834	$(AG)_8YT$	53	UBC primer dataset
UBC_842	$(GA)_8YG$	55	UBC primer dataset
UBC_840	$(GA)_8YT$	53	UBC primer dataset
UBC_850	$(GT)_8YC$	55	UBC primer dataset
UBC_851	$(GT)_8YG$	55	UBC primer dataset
UBC_857	$(AC)_8YG$	55	UBC primer dataset
UBC_881	$(GGGTG)_3$	59	UBC primer dataset
UBC_848	$(CA)_8RG$	55	UBC primer dataset
UBC_860	$(TG)_8RA$	53	UBC primer dataset

Y=T or C; R=G

Table S4. Characteristics of Alvito soil.

**Parameter (Unit)**	
Sand (% of soil dw)	16
Silt (% of dw)	27
Clay (% of dw)	57
Soil texture class	Clayey
pH (1:2.5 in water)	6.97
Conductivity (μS cm^4)	1788
Cation exchange capacity (CEC) [meq $(100g)^{-1}$]	38.9
Organic matter (% of dw)	3.25
Total N (g Kg^{-1})	1.7
C/N ratio	11.15
Assimilable P (Olsen's method, μg P g^{-1})	23.32
K [meq $(100g)^{-1}$]	0.48
Ca [meq $(100g)^{-1}$]	1.39
Na [meq $(100g)^{-1}$]	7.58
Mg [meq $(100g)^{-1}$]	0.22
Fe (ppm)	51.2
Mn (ppm)	24.37
Zn (ppm)	1.03
Cu (ppm)	3.1

Table S5. List of the qualitative and quantitative traits recorded for the 28 garlic accessions used in the experimental trial, with their corresponding scales of measurement and acronyms.

Morhological Trait	Acronym	Type	Unit	Formula for Calculation of quantitative traits	Description/levels of qualitative traits
Shape of mature dry bulbs	SMDB	Qualitative			1.Flat; 2.Flat globe; 3.Rhomboid; 4.Broad oval; 5.Globe; 6.Broad elliptic; 7.Ovate; 8.Spindle; 9.High top; 99.Other
Bulb shape in longitudinal section	BSLS	Qualitative			1.Transverse narrow elliptic; 2.Transverse broad elliptic; 3.Circular
Bulb shape in cross section	BSCS	Qualitative			1.Elliptic; 2.Circular
Outer skin color of compound bulb	OSCB	Qualitative			1.White; 2.Cream; 3.Beige; 4.White stripes; 5.Light violet; 6.Violet; 7.Dark Violet; 99.Other
Presence of anthocyanins bulb	PAB	Qualitative			1. Slight: 2. Intermediate; 3.Strong
Bulb distribution of cloves	BDC	Qualitative			1.Radial; 2.Non-radial
Bulb external cloves	BEC	Qualitative			1.Absent; 9.Present
Bulb structure type	BST	Qualitative			1.Regular multi-fan groups; 2.Regular two-fan groups; 3.Regular multi-cloved radial; 4.Regular quadruple; 5.Regular two-cloved; 6.Irregular
Skin color of the clove	SCC	Qualitative			1.White; 2.Yellow and light brown; 3.Brown; 4.Red; 5.Violet; 99.Other
Peeled clove color	PCC	Qualitative			1.White; 2.Yellowish
Bulb weight	W	Quantitative	grams (g)		
Bulb Equatorial diameter or horizontal diameter (width)	De	Quantitative	millimeters (mm)		
Bulb Polar diameter or longitudinal diameter (Height)	Dp	Quantitative	millimeters (mm)		
Bulb Thickness	T	Quantitative	millimeters (mm)		
Bulb Diameter geometric mean	Dgm	Quantitative	millimeters (mm)	$Dgm = (D_e*D_p*T)^{0,333}$	
Bulb Diameter arithmetic mean	Dam	Quantitative	millimeters (mm)	$Dam = (D_e+D_p+T)/3$	
Bulb surface area	SA	Quantitative	square millimetres $(mm)^2$	$SA = 0,785*D_e*D_p$	
Bulb Cross-sectional area	CSA	Quantitative	square millimetres $(mm)^2$	$CSA = 0,785*(D_e+D_p+T)^2/3$	
Bulb Sphericity index	SI	Quantitative		$D_e/\sqrt{D_p}*T$	
Bulb dry matter	DM	Quantitative	percentage	Dry weight /Fresh weigt *100	
Number of cloves per bulb	NCLBulb	Quantitative			
Clove average weight	CLW	Quantitative	milligrams (mg)		
Peeled clove color (L*)	L*	Quantitative	CIELAB space		
Peeled clove color (a*)	a*	Quantitative	CIELAB space		
Peeled clove color (b*)	b*	Quantitative	CIELAB space		
Peeled clove color (C)	C	Quantitative	CIELAB space		
Peeled clove color (h)	h	Quantitative	CIELAB space		

Table S6. SSR private alleles detected in the 35 garlic genotypes in homozygous (Hom) or heterozygous (Het) status.

Locus	Allele	Frequency	Genotype	Status
GB_ASM_040	374	0.029	AGLIONE	Hom
GB_ASM_078	240	0.029	AGLIONE	Het
Asa_24	145	0.029	AGLIONE	Hom
Asa_17	256	0.029	AGLIONE	Hom
Asa_25	247	0.029	B_MONT1	Het
	483	0.029	AGLIONE	Hom
Asa_10	94	0.029	AGLIONE	Hom
	229	0.029	R_SPA4	Hom
AS_5944	159	0.029	AGLIONE	Het
	169	0.029	AGLIONE	Het
	186	0.029	R_NUB1	Het
	192	0.029	R_NUB1	Het
	206	0.029	LAZIO_SUD	Het
AS_739	202	0.029	R_CANN1	Het
AS_589	230	0.029	AGLIONE	Hom
	240	0.029	R_NUB1	Hom
AS_987	226	0.029	R_CANN1	Het
	246	0.029	AGLIONE	Het
AS_30	240	0.029	AGLIONE	Hom
	266	0.029	LAZIO_SUD	Het
	270	0.029	R_NUB1	Het
	298	0.029	R_CANN1	Het

Table S7. ISSR private bands (alleles) detected in the 35 garlic accessions.

Locus	Frequency	Genotype
UBC_832/L7	0.029	B_MONT_1
UBC_832/L13	0.029	AGLIONE
UBC_832/L14	0.029	LAZIO_SUD
UBC_832/L16	0.029	R_SPA_2
UBC_832/L19	0.029	AGLIONE
UBC_832/L20	0.029	AGLIONE
UBC_834/L23	0.029	AGLIONE
UBC_834/L32	0.029	AGLIONE
UBC_834/L33	0.029	AGLIONE
UBC_842/L39	0.029	AGLIONE
UBC_842/L43	0.029	AGLIONE
UBC_842/L46	0.029	AGLIONE
UBC_842/L47	0.029	B_MONT1
UBC_842/L48	0.029	R_CANN1
UBC_842/L51	0.029	AGLIONE
UBC_842/L53	0.029	LAZIO_SUD
UBC_840/L56	0.029	LAZIO_SUD
UBC_840/L57	0.029	AGLIONE
UBC_840/L60	0.029	AGLIONE
UBC_840/L63	0.029	LAZIO_SUD
UBC_840/L66	0.029	LAZIO_SUD
UBC_840/L68	0.029	LAZIO_SUD
UBC_840/L69	0.029	AGLIONE
UBC_850/L71	0.029	AGLIONE
UBC_850/L73	0.029	B_MONT1
UBC_850/L76	0.029	AGLIONE
UBC_850/L78	0.029	LAZIO_SUD
UBC_850/L79	0.029	AGLIONE
UBC_851/L105	0.029	AGLIONE
UBC_851/L106	0.029	LAZIO_SUD
UBC_851/L108	0.029	LAZIO_SUD
UBC_851/L109	0.029	AGLIONE
UBC_857/L110	0.029	AGLIONE
UBC_857/L113	0.029	AGLIONE
UBC_857/L117	0.029	B_MONT1
UBC_857/L121	0.029	AGLIONE
UBC_857/L125	0.029	LAZIO_SUD
UBC_881/L126	0.029	B_MONT1
UBC_881/L129	0.029	LAZIO_SUD
UBC_881/L130	0.029	AGLIONE
UBC_881/L137	0.029	LAZIO_SUD
UBC_881/L138	0.029	B_MONT1
UBC_881/L139	0.029	LAZIO_SUD
UBC_881/L140	0.029	AGLIONE
UBC_881/L141	0.029	AGLIONE
UBC_881/L143	0.029	AGLIONE
UBC_881/L146	0.029	AGLIONE
UBC_848/L150	0.029	AGLIONE
UBC_848/L156	0.029	LAZIO_SUD
UBC_848/L165	0.029	AGLIONE
UBC_848/L167	0.029	AGLIONE
UBC_860/L171	0.029	AGLIONE
UBC_860/L173	0.029	LAZIO_SUD
UBC_860/L176	0.029	AGLIONE
UBC_860/L177	0.029	LAZIO_SUD
UBC_860/L181	0.029	AGLIONE

Table S8. Posterior membership coefficients (Q) following a STRUCTURE analysis of the 34 *A. sativum* accessions with K=2 and K=4.

Accession	K=2		K=4			
R_PROC 1	0.996	0.004	0.987	0.013	0.000	0.000
R_PROC 2	0.996	0.004	0.987	0.013	0.000	0.000
R_PROC 6	0.997	0.003	0.990	0.010	0.000	0.000
R_PROC 4	0.987	0.013	0.981	0.019	0.000	0.000
R_PROC 5	0.997	0.003	0.977	0.023	0.000	0.000
R_PROC 8	0.998	0.002	0.595	0.405	0.000	0.000
R_PROC 3	0.998	0.002	0.567	0.433	0.000	0.000
R_PROC 7	0.998	0.002	0.703	0.297	0.000	0.000
R_PROC 9	0.996	0.004	0.593	0.407	0.000	0.000
R_SULM 3	0.995	0.005	0.298	0.702	0.000	0.000
R_SULM 2	0.881	0.119	0.593	0.407	0.000	0.000
R_SULM 1	0.938	0.062	0.604	0.396	0.000	0.000
R_SPA 1	0.988	0.012	0.528	0.472	0.000	0.000
R_MAREM 1	0.918	0.082	0.492	0.508	0.000	0.000
R_SORA 1	0.976	0.024	0.385	0.615	0.000	0.000
R_SULM 4	0.980	0.020	0.294	0.706	0.000	0.000
R_CAST 3	0.998	0.002	0.068	0.932	0.000	0.000
R_CAST 6	0.998	0.002	0.065	0.935	0.000	0.000
R_CAST 7	0.998	0.002	0.018	0.982	0.000	0.000
R_CAST 4	0.999	0.001	0.009	0.991	0.000	0.000
R_CAST 11	0.998	0.002	0.036	0.964	0.000	0.000
R_CAST 5	0.999	0.001	0.023	0.977	0.000	0.000
R_CAST 10	0.999	0.001	0.022	0.978	0.000	0.000
R_CAST 2	0.998	0.002	0.010	0.990	0.000	0.000
R_CAST 9	0.998	0.002	0.014	0.986	0.000	0.000
R_CAST 1	0.120	0.880	0.000	0.000	0.996	0.004
R_CAST 8	0.150	0.850	0.000	0.000	0.989	0.011
R_CANN 1	0.089	0.911	0.000	0.000	0.527	0.473
R_NUB 1	0.099	0.901	0.000	0.000	0.633	0.367
R_SPA 2	0.150	0.850	0.000	0.000	0.987	0.013
R_SPA 3	0.049	0.951	0.000	0.000	0.996	0.004
R_SPA 4	0.007	0.993	0.000	0.000	0.993	0.007
B_MONT 1	0.003	0.997	0.000	0.000	0.012	0.880
LAZIO SUD	0.001	0.999	0.000	0.000	0.015	0.985

Table S9. SSR private alleles detected in "Aglio Rosso di Castelliri" and "Aglio Rosso di Proceno" landraces.

**Locus**	**Allele**	**Landrace**	**Frequency**	**Accession name**
GB_ASM_059	300	Proceno	0.05	R_PROC9
Asa_10	228	Proceno	0.11	R_PROC6-7
AS_11065	222	Castelliri	0.05	R_CAST6
AS_739	197	Castelliri	1.00	
AS_739	206	Castelliri	1.00	
AS_739	200	Proceno	1.00	
AS_739	209	Proceno	1.00	
AS_589	240	Castelliri	1.00	
AS_589	242	Proceno	1.00	

Table S10. ISSR private alleles and their frequencies detected in the "Aglio Rosso di Proceno" landrace. No private alleles were found in the "Aglio Rosso di Castelliri" landrace.

Locus	Landrace	Frequency	Accession name
UBC_842/L42	Proceno	0.22	R_PROC3/7/8/9
UBC_842/L48	Proceno	0.05	R_PROC9
UBC_850/L92	Proceno	0.22	R_PROC1/2/4/6
UBC_850/L94	Proceno	0.22	R_PROC1/2/4/7
UBC_857/L111	Proceno	0.27	R_PROC1/2/4/5/7
UBC_848/L151	Proceno	0.27	R_PROC1/2/4/5/7
UBC_848/L152	Proceno	0.27	R_PROC1/2/4/5/7
UBC_848/L161	Proceno	0.27	R_PROC1/2/4/5/7
UBC_848/L163	Proceno	0.27	R_PROC1/2/4/5/7

Table S11. Morphological qualitative traits of bulbs and cloves retrieved by using UPOV and IPGRI descriptors for the 28 garlic accessions. Extension of the acronyms for the qualitative traits are as indicated in Table S5.

Accession	SMDB	BSLS	BSCS	OSCB	PAB	BDC	BEC	BST	SCC	PCC
R_PROC_1	Rhomboid	Transverse broad elliptic	Circular	White	Slight	Radial	Absent	Regular two-fan groups	Violet	Yellowish
R_PROC_2	Rhomboid	Transverse broad elliptic	Circular	White	Slight	Radial	Absent	Regular two-fan groups	Violet	Yellowish
R_PROC_3	Rhomboid	Transverse broad elliptic	Circular	White	Slight	Radial	Absent	Regular two-fan groups	Violet	Yellowish
R_PROC_4	Rhomboid	Transverse broad elliptic	Circular	White	Slight	Radial	Absent	Regular two-fan groups	Violet	Yellowish
R_PROC_5	Rhomboid	Transverse broad elliptic	Circular	White	Slight	Radial	Absent	Regular two-fan groups	Violet	Yellowish
R_PROC_6	Rhomboid	Transverse broad elliptic	Circular	White	Slight	Radial	Absent	Regular two-fan groups	Violet	Yellowish
R_PROC_7	Rhomboid	Transverse broad elliptic	Circular	White	Slight	Radial	Absent	Regular two-fan groups	Violet	Yellowish
R_PROC_8	Rhomboid	Transverse broad elliptic	Circular	White	Slight	Radial	Absent	Regular two-fan groups	Violet	Yellowish
R_PROC_9	Rhomboid	Transverse broad elliptic	Circular	White	Slight	Radial	Absent	Regular two-fan groups	Violet	Yellowish
R_CAST_1	Rhomboid	Transverse broad elliptic	Circular	White	Slight	Radial	Absent	Regular two-fan groups	Violet	Yellowish
R_CAST_2	Rhomboid	Transverse broad elliptic	Circular	White	Slight	Radial	Absent	Regular two-fan groups	Violet	Yellowish
R_CAST_3	Rhomboid	Transverse broad elliptic	Circular	White	Slight	Radial	Absent	Regular two-fan groups	Violet	Yellowish
R_CAST_4	Rhomboid	Transverse broad elliptic	Circular	White	Slight	Radial	Absent	Regular two-fan groups	Violet	Yellowish
R_CAST_5	Rhomboid	Transverse broad elliptic	Circular	White	Slight	Radial	Absent	Regular two-fan groups	Violet	Yellowish
R_CAST_6	Rhomboid	Transverse broad elliptic	Circular	White	Slight	Radial	Absent	Regular two-fan groups	Violet	Yellowish
R_CAST_7	Rhomboid	Transverse broad elliptic	Circular	White	Slight	Radial	Absent	Regular two-fan groups	Violet	Yellowish
R_CAST_8	Rhomboid	Transverse broad elliptic	Circular	White	Slight	Radial	Absent	Regular two-fan groups	Violet	Yellowish
R_CAST_9	Rhomboid	Transverse broad elliptic	Circular	White	Slight	Radial	Absent	Regular two-fan groups	Violet	Yellowish
R_CAST_10	Rhomboid	Transverse broad elliptic	Circular	White	Slight	Radial	Absent	Regular two-fan groups	Violet	Yellowish
R_CAST_11	Rhomboid	Transverse broad elliptic	Circular	White	Slight	Radial	Absent	Regular two-fan groups	Violet	Yellowish
R_SULM_2	Rhomboid	Transverse broad elliptic	Circular	White/Cream	Slight	Radial	Absent	Regular two-fan groups	Violet	Yellowish
R_SULM_4	Rhomboid	Transverse broad elliptic	Circular	White/Cream	Slight	Radial	Absent	Regular two-fan groups	Violet	Yellowish
R_SULM_3	Rhomboid	Transverse broad elliptic	Circular	White	Slight	Radial	Absent	Regular two-fan groups	Violet	Yellowish
R_SORA_1	Rhomboid	Transverse broad elliptic	Circular	White	Slight	Radial	Absent	Regular two-fan groups	Violet	Yellowish
R_CANN_1	Rhomboid	Transverse broad elliptic	Circular	White	Slight	Radial	Absent	Regular two-fan groups	Violet	Yellowish
R_MAREM_1	Rhomboid	Transverse broad elliptic	Circular	White	Slight	Radial	Absent	Regular two-fan groups	Violet	Yellowish
R_SPA_2	Rhomboid	Transverse broad elliptic	Circular	White	Intermediate	Radial	Absent	Regular two-fan groups	Violet	Yellowish
R_SPA_4	Rhomboid	Transverse broad elliptic	Circular	White	Intermediate	Radial	Absent	Regular two-fan groups	Violet	Yellowish

Table S12. Differences in 12 morphological quantitative traits of bulbs and cloves (means of four measurements) for the 28 garlic accessions. Refer to Table S5 for acronyms of quantitative traits. Mean values and standard deviations in columns with different letters are significantly different at $p<0.05$ according to Tukey's HSD test; *** indicates significant difference at $p<0.001$ following ANOVA analysis; ns = not significant; CV= coefficient of variation expressed a percentage.

Accession	W	De	Dp	T	Dgm	Dam	SA	CSA	SI	NCLBulb	CLW	DM
R_PROC_1	23.40 ± 2.20 $^{b-g}$	40.39 ± 1.53 $^{a-f}$	32.73 ± 1.30 $^{a-c}$	36.01 ± 1.86 $^{a-d}$	34.96 ± 1.43 $^{a-d}$	36.38 ± 1.50 $^{a-d}$	1038.66 ± 77.05 $^{a-c}$	3120.13 ± 256.45 $^{a-d}$	1.18 ± 0.02 $^{a-b}$	11.5 ± 2.08 $^{a-b}$	2.06 ± 0.21 $^{a-e}$	39.75 ± 0.93 $^{g-h}$
R_PROC_2	22.25 ± 2.69 $^{b-i}$	39.58 ± 1.98 $^{a-f}$	32.37 ± 1.67 $^{a-c}$	34.91 ± 2.29 $^{a-d}$	34.25 ± 1.81 $^{a-d}$	35.62 ± 1.90 $^{a-d}$	1007.58 ± 99.96 $^{a-c}$	2994.66 ± 327.50 $^{a-d}$	1.18 ± 0.02 $^{a-b}$	11 ± 1.41 $^{a-b}$	2.05 ± 0.35 $^{a-f}$	38.39 ± 0.89 $^{h-i}$
R_PROC_3	17.08 ± 3.28 $^{h-i}$	36.04 ± 3.36 $^{b-f}$	28.39 ± 2.42 ^{c}	31.08 ± 4.37 $^{c-d}$	30.59 ± 3.20 ^{d}	31.84 ± 3.36 ^{d}	807.80 ± 141.99 ^{c}	2406.62 ± 502.78 ^{d}	1.22 ± 0.04 ^{a}	9.75 ± 0.96 $^{a-b}$	1.74 ± 0.18 $^{d-g}$	43.45 ± 1.00 $^{a-d}$
R_PROC_4	24.91 ± 2.65 $^{a-c}$	41.39 ± 1.62 $^{a-e}$	33.31 ± 1.92 $^{a-c}$	37.24 ± 2.17 $^{a-c}$	35.84 ± 1.81 $^{a-d}$	37.31 ± 1.88 $^{a-d}$	1083.94 ± 101.86 $^{a-c}$	3284.62 ± 326.09 $^{a-d}$	1.18 ± 0.03 $^{a-b}$	11.25 ± 1.71 $^{a-b}$	2.23 ± 0.11 $^{a-b}$	38.87 ± 0.84 $^{h-i}$
R_PROC_5	22.97 ± 2.07 $^{b-h}$	39.35 ± 1.09 $^{a-f}$	31.77 ± 1.84 $^{a-c}$	34.98 ± 1.19 $^{a-d}$	33.99 ± 1.25 $^{a-d}$	35.37 ± 1.27 $^{a-d}$	982.44 ± 82.44 $^{a-c}$	2948.50 ± 211.50 $^{a-d}$	1.18 ± 0.02 $^{a-b}$	10.75 ± 1.26 $^{a-b}$	2.14 ± 0.08 $^{a-d}$	40.05 ± 1.17 $^{f-h}$
R_PROC_6	23.5 ± 2.19 $^{b-f}$	40.14 ± 2.22 $^{a-f}$	32.01 ± 1.37 $^{a-c}$	36.00 ± 2.51 $^{a-d}$	34.62 ± 1.83 $^{a-d}$	36.05 ± 1.94 $^{a-d}$	1009.68 ± 90.59 $^{a-c}$	3066.51 ± 327.80 $^{a-d}$	1.18 ± 0.03 $^{a-b}$	11.25 ± 0.50 $^{a-b}$	2.09 ± 0.12 $^{a-e}$	40.06 ± 1.33 $^{f-h}$
R_PROC_7	17.78 ± 1.88 $^{f-i}$	35.66 ± 2.19 $^{d-f}$	29.03 ± 2.56 $^{b-c}$	30.6 ± 1.83 ^{d}	30.56 ± 2.03 ^{d}	31.77 ± 2.11 ^{d}	815.48 ± 116.55 ^{c}	2384.20 ± 313.90 ^{d}	1.20 ± 0.05 $^{a-b}$	9.75 ± 0.96 $^{a-b}$	1.82 ± 0.07 $^{c-g}$	43.09 ± 0.97 $^{d-f}$
R_PROC_8	16.27 ± 1.25 ^{i}	35.50 ± 1.95 $^{d-f}$	30.15 ± 1.59 $^{b-c}$	31.41 ± 1.76 $^{c-d}$	31.16 ± 1.46 $^{b-d}$	32.35 ± 1.54 $^{b-d}$	841.20 ± 79.65 $^{b-c}$	2468.85 ± 229.75 $^{c-d}$	1.15 ± 0.04 $^{a-b}$	9.25 ± 0.50 ^{b}	1.76 ± 0.14 $^{c-g}$	45.13 ± 1.16 $^{a-c}$
R_PROC_9	16.9 ± 2.55 $^{h-i}$	34.90 ± 2.42 ^{f}	30.72 ± 2.13 $^{b-c}$	31.18 ± 1.90 $^{c-d}$	31.11 ± 2.01 $^{b-d}$	32.26 ± 2.10 $^{b-d}$	844.34 ± 114.05 $^{b-c}$	2459.44 ± 319.34 $^{c-d}$	1.13 ± 0.02 ^{b}	9.25 ± 0.96 ^{b}	1.84 ± 0.28 $^{b-g}$	44.15 ± 0.99 $^{a-d}$
R_CAST_1	25.00 ± 2.79 $^{a-b}$	41.79 ± 2.50 $^{a-d}$	33.54 ± 1.39 $^{a-c}$	37.88 ± 2.60 $^{a-b}$	36.24 ± 1.97 $^{a-c}$	37.74 ± 2.10 $^{a-c}$	1102.26 ± 108.47 $^{a-c}$	3361.45 ± 365.95 $^{a-c}$	1.17 ± 0.01 $^{a-b}$	12.25 ± 1.71 $^{a-b}$	2.05 ± 0.11 $^{a-f}$	40.07 ± 0.63 $^{e-h}$
R_CAST_2	17.85 ± 1.46 $^{f-i}$	35.85 ± 1.40 $^{c-f}$	30.74 ± 1.67 $^{b-c}$	32.24 ± 2.17 $^{b-d}$	31.74 ± 1.68 $^{b-d}$	32.94 ± 1.74 $^{b-d}$	866.46 ± 80.37 $^{b-c}$	2561.02 ± 269.25 $^{b-d}$	1.14 ± 0.02 $^{a-b}$	10.5 ± 0.58 $^{a-b}$	1.70 ± 0.08 $^{e-g}$	44.05 ± 0.82 $^{a-d}$
R_CAST_3	17.3 ± 1.72 $^{g-i}$	36.07 ± 3.25 $^{b-f}$	30.03 ± 1.93 $^{b-c}$	31.35 ± 2.88 $^{c-d}$	31.27 ± 2.52 $^{b-d}$	32.48 ± 2.66 $^{b-d}$	854.06 ± 130.23 $^{b-c}$	2497.55 ± 406.37 $^{b-d}$	1.17 ± 0.02 $^{a-b}$	10.5 ± 1.29 $^{a-b}$	1.65 ± 0.08 $^{f-g}$	44.58 ± 0.81 $^{a-c}$
R_CAST_4	17.17 ± 1.96 $^{h-i}$	35.10 ± 3.10 $^{e-f}$	29.67 ± 1.95 $^{b-c}$	31.11 ± 3.38 $^{c-d}$	30.79 ± 2.67 $^{c-d}$	31.96 ± 2.81 $^{c-d}$	820.86 ± 125.21 ^{c}	2418.89 ± 420.99 ^{d}	1.16 ± 0.01 $^{a-b}$	9.75 ± 1.50 $^{a-b}$	1.77 ± 0.10 $^{c-g}$	45.40 ± 1.46 $^{a-b}$
R_CAST_5	17.85 ± 1.80 $^{f-i}$	36.25 ± 2.86 $^{b-f}$	30.82 ± 2.01 $^{a-c}$	32.02 ± 3.03 $^{b-d}$	31.81 ± 2.48 $^{b-d}$	33.03 ± 2.60 $^{b-d}$	880.00 ± 122.98 $^{b-c}$	2580.80 ± 401.09 $^{b-d}$	1.15 ± 0.03 $^{a-b}$	9.5 ± 1.29 $^{a-b}$	1.89 ± 0.08 $^{b-g}$	45.23 ± 1.39 $^{a-c}$
R_CAST_6	17.87 ± 2.16 $^{e-i}$	36.75 ± 3.41 $^{b-f}$	30.27 ± 2.91 $^{b-c}$	32.77 ± 2.88 $^{b-d}$	32.01 ± 2.91 $^{b-d}$	33.26 ± 3.05 $^{b-d}$	879.16 ± 167.70 $^{b-c}$	2622.01 ± 486.58 $^{b-d}$	1.17 ± 0.01 $^{a-b}$	11.5 ± 1.73 $^{a-b}$	1.56 ± 0.15 ^{g}	44.55 ± 1.13 $^{a-d}$
R_CAST_7	16.9 ± 1.66 $^{h-i}$	35.12 ± 2.54 $^{e-f}$	29.46 ± 1.31 $^{b-c}$	31.3 ± 2.68 $^{c-d}$	30.79 ± 2.03 $^{c-d}$	31.96 ± 2.15 $^{c-d}$	814.07 ± 93.71 ^{c}	2413.80 ± 323.30 ^{d}	1.16 ± 0.02 $^{a-b}$	10.25 ± 0.96 $^{a-b}$	1.65 ± 0.15 $^{f-g}$	46.38 ± 1.12 ^{a}
R_CAST_8	24.45 ± 3.01 $^{a-d}$	41.10 ± 3.69 $^{a-f}$	33.02 ± 2.04 $^{a-c}$	37.14 ± 2.82 $^{a-c}$	35.62 ± 2.62 $^{a-d}$	37.08 ± 2.80 $^{a-d}$	1068.91 ± 161.23 $^{a-c}$	3251.61 ± 490.67 $^{a-d}$	1.17 ± 0.03 $^{a-b}$	11.75 ± 0.96 $^{a-b}$	2.08 ± 0.10 $^{a-e}$	38.98 ± 1.19 $^{g-i}$
R_CAST_9	18.47 ± 2.29 $^{d-i}$	36.44 ± 1.19 $^{b-f}$	30.56 ± 2.71 $^{b-c}$	32.83 ± 2.02 $^{b-d}$	32.04 ± 1.93 $^{b-d}$	33.28 ± 1.97 $^{b-d}$	876.08 ± 106.18 $^{b-c}$	2614.78 ± 309.17 $^{b-d}$	1.15 ± 0.05 $^{a-b}$	10.25 ± 1.50 $^{a-b}$	1.81 ± 0.11 $^{c-g}$	45.14 ± 1.21 $^{a-c}$
R_CAST_10	18.8 ± 2.03 $^{c-i}$	37.14 ± 2.11 $^{b-f}$	29.83 ± 2.92 $^{b-c}$	33.23 ± 1.72 $^{b-d}$	32.11 ± 2.18 $^{b-d}$	33.40 ± 2.24 $^{b-d}$	873.33 + 134.75 $^{b-c}$	2635.73 ± 353.10 $^{b-d}$	1.18 ± 0.02 $^{a-b}$	11.5 ± 1.29 $^{a-b}$	1.64 ± 0.13 ^{g}	44.28 ± 1.12 $^{a-d}$
R_CAST_11	17.42 ± 2.03 $^{f-i}$	35.74 ± 2.66 $^{d-f}$	29.49 ± 1.50 $^{b-c}$	31.99 ± 2.11 $^{b-d}$	31.2 ± 1.91 $^{b-d}$	32.41 ± 2.03 $^{b-d}$	829.43 ± 101.95 $^{b-c}$	2480.50 ± 313.94 $^{b-d}$	1.16 ± 0.03 $^{a-b}$	10.75 ± 0.96 $^{a-b}$	1.62 ± 0.15 ^{g}	44.24 ± 0.91 $^{a-d}$
R_SULM_2	23.52 ± 2.88 $^{b-f}$	39.79 ± 2.43 $^{a-f}$	32.75 ± 2.55 $^{a-c}$	36.11 ± 2.78 $^{a-d}$	34.83 ± 2.46 $^{a-d}$	36.22 ± 2.57 $^{a-d}$	1026.31 ± 140.38 $^{a-c}$	3100.29 ± 438.67 $^{a-d}$	1.16 ± 0.03 $^{a-b}$	10.25 ± 1.26 $^{a-b}$	2.30 ± 0.04 ^{a}	43.27 ± 1.07 $^{a-e}$
R_SULM_4	24.02 ± 1.18 $^{b-e}$	40.61 ± 1.62 $^{a-f}$	32.53 ± 1.12 $^{a-c}$	35.82 ± 1.11 $^{a-d}$	34.89 ± 1.19 $^{a-d}$	36.32 ± 1.26 $^{a-d}$	1037.95 ± 74.50 $^{a-c}$	3109.22 ± 213.39 $^{a-d}$	1.19 ± 0.02 $^{a-b}$	10.5 ± 0.58 $^{a-b}$	2.29 ± 0.19 ^{a}	42.12 ± 1.13 $^{c-g}$
R_SULM_3	25.6 ± 2.16 $^{a-d}$	42.16 ± 1.61 $^{a-c}$	34.04 ± 1.35 $^{a-b}$	37.84 ± 2.23 $^{a-b}$	36.51 ± 1.59 $^{a-b}$	38.01 ± 1.67 $^{a-b}$	1127.57 ± 86.39 $^{a-b}$	3407.18 ± 298.30 $^{a-b}$	1.18 ± 0.03 $^{a-b}$	11 ± 1.15 $^{a-b}$	2.33 ± 0.11 ^{a}	44.84 ± 1.30 $^{a-c}$
R_SORA_1	22.18 ± 2.00 $^{b-i}$	39.32 ± 1.42 $^{a-f}$	31.5 ± 1.60 $^{a-c}$	35.08 ± 1.70 $^{a-d}$	33.92 ± 1.49 $^{a-d}$	35.30 ± 1.55 $^{a-d}$	973.34 ± 84.95 $^{a-c}$	2938.49 ± 261.52 $^{a-d}$	1.18 ± 0.02 $^{a-b}$	10.25 ± 0.50 $^{a-b}$	2.16 ± 0.10 $^{a-c}$	43.03 ± 1.17 $^{b-f}$
R_CANN_1	30.3 ± 3.25 ^{a}	44.88 ± 1.73 ^{a}	36.15 ± 2.74 ^{a}	39.78 ± 0.90 ^{a}	38.65 ± 1.70 ^{a}	40.27 ± 1.75 ^{a}	1276.04 ± 141.81 ^{a}	3824.27 ± 328.94 ^{a}	1.18 ± 0.02 $^{a-b}$	12.75 ± 0.96 ^{a}	2.38 ± 0.23 ^{a}	35.99 ± 1.22 ^{i}
R_MAREM_1	17.25 ± 2.05 $^{g-i}$	36.46 ± 2.36 $^{b-f}$	29.76 ± 1.50 $^{b-c}$	32.79 ± 1.52 $^{b-d}$	31.75 ± 1.37 $^{b-d}$	33.01 ± 1.45 $^{b-d}$	853.23 ± 87.72 $^{b-c}$	2569.20 ± 226.19 $^{b-d}$	1.17 ± 0.07 $^{a-b}$	9.25 ± 0.96 ^{b}	1.86 ± 0.08 $^{b-g}$	42.15 ± 1.02 $^{c-g}$
R_SPA_2	25.15 ± 2.61 $^{a-b}$	41.35 ± 2.43 $^{a-e}$	33.64 ± 2.22 $^{a-c}$	37.37 ± 2.42 $^{a-c}$	35.99 ± 2.24 $^{a-d}$	37.46 ± 2.34 $^{a-d}$	1095.16 ± 134.45 $^{a-c}$	3313.47 ± 411.65 $^{a-d}$	1.17 ± 0.01 $^{a-b}$	11.75 ± 1.50 $^{a-b}$	2.15 ± 0.08 $^{a-c}$	41.34 ± 0.90 $^{d-h}$
R_SPA_4	25.92 ± 1.72 $^{a-b}$	42.23 ± 1.17 $^{a-b}$	33.53 ± 1.04 $^{a-c}$	37.91 ± 1.58 $^{a-b}$	36.38 ± 1.18 $^{a-b}$	37.89 ± 1.24 $^{a-b}$	1112.33 ± 64.26 $^{a-c}$	3384.12 ± 221.84 $^{a-c}$	1.18 ± 0.01 $^{a-b}$	12.25 ± 0.96 $^{a-b}$	2.12 ± 0.06 $^{a-d}$	43.42 ± 0.96 $^{a-d}$
CV	20,45	8,94	7,97	9,83	8,62	8,73	16,5	17,38	2,63	13,26	14,02	6,52
P	***	***	***	***	***	***	***	***	ns	***	***	***

Table S13. Pearson correlation coefficients of morphological quantitative traits of bulbs and cloves. Refer to Table S5 for acronyms of quantitative traits. ** Correlation significant at *p*<0.01. * Correlation significant at *p*<0.05.

	**W**	**De**	**Dp**	**T**	**Dgm**	**Dam**	**SA**	**CSA**	**SI**	**NCLBulb**	**CLW**	**DM**
**W**	1.000											
**De**	0.991**	1.000										
**Dp**	0.955**	0.947**	1.000									
**T**	0.980**	0.988**	0.954**	1.000								
**Dgm**	0.988**	0.992**	0.976**	0.995**	1.000							
**Dam**	0.989**	0.993**	0.974**	0.995**	1.000	1.000						
**SA**	0.988**	0.989**	0.983**	0.983**	0.996**	0.996**	1.000					
**CSA**	0.989**	0.993**	0.974**	0.993**	0.999**	0.999**	0.998**	1.000				
**SI**	0.354	0.392	0.106	0.284	0.275	0.284	0.273	0.287	1.000			
**NCLBulb**	0.774**	0.788**	0.723**	0.793**	0.781**	0.782**	0.774**	0.786**	0.309	1.000		
**CLW**	0.894**	0.872**	0.863**	0.856**	0.874**	0.874**	0.874**	0.870**	0.288	0.473*	1.000	
**DM**	-0.740**	-0.738**	-0.683**	-0.705**	-0.718**	-0.720**	-0.724**	-0.721**	-0.388	-0.600**	-0.635**	1.000

Table S14. Differences in color parameters of peeled cloves among the 28 garlic accessions expressed as average value (from 16 measurements) of the different components according to the CIELab scale. Mean values and standard deviations in columns with different letters are significantly different at $p< 0.05$ according to Tukey's HSD test; *** indicates significant difference at $p<0.001$ following ANOVA analysis; CV= coefficient of variation expressed a percentage.

Accession	L*	a*	b*	C	H
R_PROC_1	71.78 ± 1.06 [b]	-1.56 ± 0.06 [e-j]	24.95 ± 0.77 [a-d]	25.00 ± 0.78 [a-d]	93.57 ± 0.06 [e-h]
R_PROC_2	71.00 ± 1.13 [b]	-1.58 ± 0.09 [f-k]	24.83 ± 0.66 [a-d]	24.88 ± 0.65 [a-d]	93.64 ± 0.22 [d-g]
R_PROC_3	72.92 ± 0.79 [b]	-1.50 ± 0.14 [d-i]	24.54 ± 0.70 [a-e]	24.58 ± 0.70 [a-e]	93.51 ± 0.25 [e-h]
R_PROC_4	72.83 ± 1.48 [b]	-1.63 ± 0.17 [g-l]	27.11 ± 1.23 [a]	27.16 ± 1.23 [a]	93.45 ± 0.41 [e-h]
R_PROC_5	72.93 ± 1.23 [b]	-1.82 ± 0.15 [i-l]	27.18 ± 3.94 [a]	27.25 ± 3.93 [a]	93.91 ± 0.78 [c-f]
R_PROC_6	72.13 ± 0.81 [b]	-1.58 ± 0.17 [f-k]	25.61 ± 1.55 [a-b]	25.66 ± 1.54 [a-b]	93.54 ± 0.43 [e-h]
R_PROC_7	72.82 ± 1.14 [b]	-1.65 ± 0.30 [h-l]	24.78 ± 0.95 [a-d]	24.83 ± 0.96 [a-d]	93.79 ± 0.57 [d-f]
R_PROC_8	72.87 ± 1.00 [b]	-1.84 ± 0.23 [i-l]	26.93 ± 2.94 [a]	27.00 ± 2.92 [a]	93.97 ± 0.81 [b-f]
R_PROC_9	72.79 ± 0.69 [b]	-1.77 ± 0.32 [i-l]	25.11 ± 1.02 [a-c]	25.17 ± 1.00 [a-c]	94.05 ± 0.78 [a-e]
R_CAST_1	71.26 ± 0.90 [b]	-1.19 ± 0.16 [b-d]	21.24 ± 0.94 [e-f]	21.27 ± 0.94 [e-f]	93.21 ± 0.45 [e-h]
R_CAST_2	79.14 ± 1.16 [a]	-1.26 ± 0.06 [b-f]	22.29 ± 1.01 [b-f]	22.38 ± 1.00 [b-f]	93.25 ± 0.23 [e-h]
R_CAST_3	77.51 ± 0.37 [a]	-1.24 ± 0.23 [b-f]	20.59 ± 1.95 [f]	20.63 ± 1.94 [f]	93.50 ± 0.83 [e-h]
R_CAST_4	78.37 ± 1.00 [a]	-1.29 ± 0.18 [b-h]	22.17 ± 0.66 [c-f]	22.21 ± 0.66 [c-f]	93.32 ± 0.42 [e-h]
R_CAST_5	77.72 ± 1.00 [a]	-1.27 ± 0.08 [b-g]	22.42 ± 0.53 [b-f]	22.46 ± 0.53 [b-f]	93.23 ± 0.19 [e-h]
R_CAST_6	77.48 ± 1.17 [a]	-1.25 ± 0.18 [b-f]	22.86 ± 0.48 [b-f]	22.90 ± 0.48 [b-f]	93.12 ± 0.47 [e-h]
R_CAST_7	76.46 ± 0.33 [a]	-1.19 ± 0.16 [b-d]	22.56 ± 2.38 [b-f]	22.59 ± 2.38 [b-f]	93.03 ± 0.27 [e-h]
R_CAST_8	71.87 ± 1.44 [b]	-1.07 ± 0.20 [a-c]	22.79 ± 0.73 [b-f]	22.82 ± 0.72 [b-f]	92.69 ± 0.55 [g-i]
R_CAST_9	77.04 ± 0.52 [a]	-1.00 ± 0.14 [a-c]	21.69 ± 1.07 [d-f]	21.71 ± 1.07 [d-f]	92.65 ± 0.35 [g-i]
R_CAST_10	76.70 ± 0.47 [a]	-1.20 ± 1.00 [b-e]	22.46 ± 1.20 [b-f]	22.49 ± 1.21 [b-f]	93.07 ± 0.19 [e-h]
R_CAST_11	77.96 ± 3.14 [a]	-1.19 ± 0.12 [b-d]	22.86 ± 0.53 [b-f]	22.89 ± 0.53 [b-f]	92.99 ± 0.29 [f-i]
R_SULM_2	72.50 ± 1.44 [b]	-1.89 ± 0.13 [j-l]	21.63 ± 0.73 [d-f]	21.71 ± 0.73 [d-f]	95.00 ± 0.29 [a]
R_SULM_4	71.83 ± 0.44 [b]	-1.96 ± 0.11 [l]	22.70 ± 2.55 [b-f]	22.78 ± 2.55 [b-f]	94.97 ± 0.51 [a-b]
R_SULM_3	72.08 ± 0.47 [b]	-1.94 ± 1.00 [k-l]	22.75 ± 0.21 [b-f]	22.84 ± 0.21 [b-f]	94.88 ± 0.22 [a-c]
R_SORA_1	71.37 ± 0.61 [b]	-1.76 ± 0.08 [i-l]	21.82 ± 1.61 [c-f]	21.89 ± 1.61 [c-f]	94.63 ± 0.33 [a-d]
R_CANN_1	65.84 ± 3.90 [c]	-0.08 ± 0.21 [a]	22.93 ± 2.77 [b-f]	22.94 ± 2.77 [b-f]	92.10 ± 0.57 [i]
R_MAREM_1	79.22 ± 0.86 [a]	-1.09 ± 0.16 [a-c]	22.93 ± 1.20 [b-f]	22.95 ± 1.20 [b-f]	92.72 ± 0.42 [g-i]
R_SPA_2	71.48 ± 0.62 [b]	-1.36 ± 0.17 [c-h]	21.85 ± 1.20 [c-f]	21.90 ± 1.19 [c-f]	93.57 ± 0.51 [e-h]
R_SPA_4	70.23 ± 0.89 [b]	-1.95 ± 0.09 [a-b]	20.93 ± 0.50 [f]	20.96 ± 0.50 [f]	92.59 ± 0.23 [h-i]
CV	4.44	25.03	7.96	7.97	0.78
P	***	***	***	***	***

Table S15. Total eigenvalues, relative and cumulative proportion of total variance explained by each component and link of the first two PCs with the morphological and color traits of bulbs and cloves. Refer to Table S5 for acronyms of morphological traits.

Component	Total eigenvalue	Variance explained (%)	Cumulative variance (%)	Larger correlation with main traits
1	10.5838	62.258	62.258	W, De, Dp, T, Dgm, Dam, SA, CSA, SI, NCLBulb, CLW , DM, L*
2	2.8335	16.668	78.926	a*, b*, C, h
3	1.8261	10.741	89.667	
4	0.956	5.623	95.29	
5	0.3153	1.855	97.145	
6	0.2511	1.477	98.622	
7	0.1828	1.076	99.698	
8	0.0246	0.145	99.842	
9	0.0236	0.139	99.981	
10	0.0015	0.009	99.99	
11	0.001	0.006	99.996	
12	0.0003	0.002	99.998	
13	0.0002	0.001	99.999	
14	0.0001	0.001	100	

Table S16. The correlation between genetic and phenotypic (antioxidant activity and total phenolics) distance matrices using the Mantel test. AO: antioxidant activity. TPC: total phenolic content.

[a] r(xy) correlation value between the genetic (x) and phenotypic (y) matrices.
[b] p-value calculated using the distribution of r (xy) estimated from 9999 permutations. $p<0.05$ was considered significant.

	GD *vs* AO&TPC	GD *vs* AO	GD *vs* TPC
r (xy) [a]	-0.02	0.004	-0.04
p-value [b]	0.60	0.40	0.53
